# Identifying the Genes Responsible for Iron-Limited Condition in* Riemerella anatipestifer* CH-1 through RNA-Seq-Based Analysis

**DOI:** 10.1155/2017/8682057

**Published:** 2017-04-30

**Authors:** MaFeng Liu, Mi Huang, DeKang Zhu, MingShu Wang, RenYong Jia, Shun Chen, KunFeng Sun, Qiao Yang, Ying Wu, Francis Biville, AnChun Cheng

**Affiliations:** ^1^Institute of Preventive Veterinary Medicine, Sichuan Agricultural University, Chengdu, Sichuan 611130, China; ^2^Research Center of Avian Disease, College of Veterinary Medicine of Sichuan Agricultural University, Chengdu, Sichuan 611130, China; ^3^Key Laboratory of Animal Disease and Human Health of Sichuan Province, Chengdu, Sichuan 611130, China; ^4^Unité des Infections Bactériennes Invasives, Département Infection et Epidémiologie, Institut Pasteur, Paris, France

## Abstract

One of the important elements for most bacterial growth is iron, the bioavailability of which is limited in hosts.* Riemerella anatipestifer *(*R. anatipestifer*, RA), an important duck pathogen, requires iron to live. However, the genes involved in iron metabolism and the mechanisms of iron transport are largely unknown. Here, we investigated the transcriptomic effects of iron limitation condition on* R. anatipestifer *CH-1 using the RNA-Seq and RNA-Seq-based analysis. Data analysis revealed genes encoding functions related to iron homeostasis, including a number of putative TonB-dependent receptor systems, a HmuY-like protein-dependent hemin (an iron-containing porphyrin) uptake system, a Feo system, a gene cluster related to starch utilization, and genes encoding hypothetical proteins that were significantly upregulated in response to iron limitation. Compared to the number of upregulated genes, more genes were significantly downregulated in response to iron limitation. The downregulated genes mainly encoded a number of outer membrane receptors, DNA-binding proteins, phage-related proteins, and many hypothetical proteins. This information suggested that RNA-Seq-based analysis in iron-limited medium is an effective and fast method for identifying genes involved in iron uptake in* R. anatipestifer *CH-1.

## 1. Introduction

*Riemerella anatipestifer* (*R. anatipestifer*, RA) is a Gram-negative bacterium that belongs to the family Flavobacteriaceae in the rRNA superfamily V [1].* R. anatipestifer* infection causes disease in ducks, geese, chickens, turkeys, and other waterfowl and birds [2]. The disease presents as an acute or chronic septicemia characterized by meningitis, fibrinous pericarditis, perihepatitis, and other symptoms [3]. The disease causes increased mortality and decreased weight and is estimated to result in huge economic losses to the duck industry each year worldwide. At present, at least 21 serotypes of* R. anatipestifer* have been identified in the world [2, 4].

Iron is one of the most important elements for bacterial growth, as it is an essential cofactor in many important enzymes involved in energy metabolism and nucleotide synthesis [5]. Iron is the second most abundant metal on earth, but it exists primarily in the insoluble ferric oxide form under aerobic conditions, which is not available for bacterial growth [5]. Inside the host, most iron is bound to iron-binding proteins, such as ferritin, transferrin, and lactoferrin. Iron could also be included in the heme of hemoproteins (the terminology “heme” was used when talking about hemoproteins) [6]. Bacteria employ various mechanisms to capture iron from the outside [7]. One of these mechanisms is the secretion of a small molecular compound, a siderophore, which sequesters iron from the outside environment by high-affinity interactions [8]. Then, iron-bound siderophores are taken up by the bacteria through specific siderophore receptors and transport systems [9]. Alternatively, some pathogens have specific cell surface receptors that bind hemin (the terminology “hemin” was used when talking about iron and protoporphyrin ring source) or hemoprotein and transport hemin to the cell or secreted hemophores that capture hemin from host hemoproteins and then deliver hemin to bacterial surface receptors [10]. Therefore, iron-limited conditions are able to prompt most bacteria to upregulate the expression of genes related to iron/hemin uptake, such as iron/hemin transporters and siderophore biosynthetic enzymes [11–13].

*R. anatipestifer *requires iron and hemin to survive [14]. Genome analysis has shown that* R. anatipestifer* codes for a large number of TonB-dependent receptors, a TonB family protein, two sets of TonB complexes, and an FeoAB system [15]. In a previous study, we demonstrated that TonB1 and TonB2 are involved in hemin uptake by* R. anatipestifer* ATCC11845 [14]. Moreover some hemin binding proteins were detected in* R. anatipestifer* CH-1 [16]. However, other genes involved in iron/hemin uptake by* R. anatipestifer* are largely unknown. In this study, we analyzed the global transcriptomic changes in* R. anatipestifer* CH-1 under iron-limited conditions. Here, we observed wide-ranging effects on the transcripts of iron-related genes of* R. anatipestifer* CH-1 and identified some new genes involved in iron/hemin uptake.

## 2. Materials and Methods

### 2.1. Bacterial Strains and Growth Conditions

**Figure 1:**
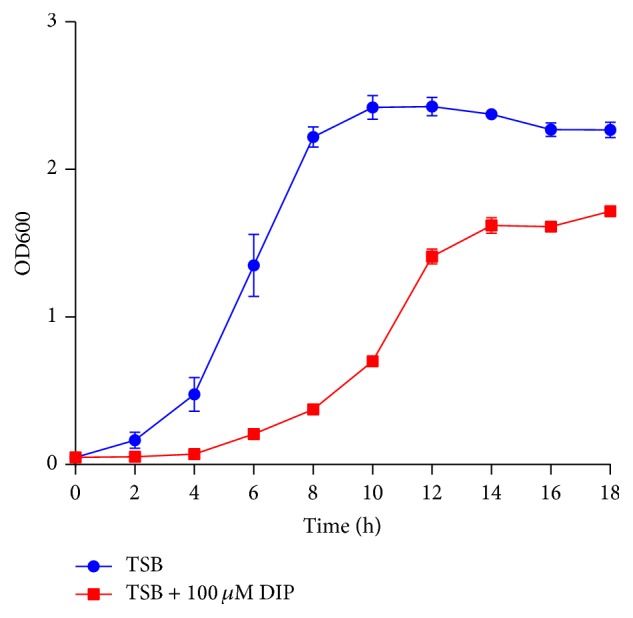
Growth curves of* R. anatipestifer* CH-1 in iron-limited and iron-replete media. Optical densities at a wavelength of 600 nm were taken from the 2nd to the 18th hours at intervals of 2 hours. Measurements were performed on triplicate samples.

For transcriptome analyses,* R. anatipestifer *CH-1 was grown in tryptone soy broth (TSB) medium (Sigma, China) as the iron-replete condition, while the iron-limited condition was TSB supplemented with 100 *μ*M iron chelator 2,2′-dipyridyl (Dip). The bacteria were cultured at 37°C with shaking at 180 rpm/min. Then, they were harvested at OD600 = 0.6 for iron-limited cultures and OD600 = 1.1 for iron-replete cultures (Figure 1).

### 2.2. RNA-Seq

Total RNA extraction was performed using the RNeasy Protect Bacteria Mini Kit (QIAGEN, Cat. number 74524) using the protocol described by Liu et al. [17]. A total amount of 3 *μ*g RNA per sample was used for the RNA sample preparations. RNA quantification, library preparation, and sequencing were performed at Beijing Novogene as described elsewhere. Then the clean data were obtained by removing reads containing adapter, reads containing ploy-N, and low-quality reads from raw data [18]. The high-quality reads obtained for each library were shown in Table 1. Then the* R. anatipestifer *CH-1 genome (CP003787.1) and gene model annotation files were downloaded from genome website (https://www.ncbi.nlm.nih.gov/nuccore/CP003787.1) directly, using Bowtie2-2.2.3 to build index and align clean reads of the* R. anatipestifer* CH-1 genome [19].

### 2.3. Real-Time PCR Validation of RNA-Seq

The differential expression of selected genes was validated by quantitative reverse transcription polymerase chain reaction (qRT-PCR) using the SYBR green-based detection system on a CFX Connect® Real-Time PCR Detection System (Bio-Rad Laboratories, Hercules, CA) using the KAPA SYBR® FAST qPCR kit (KAPABIOSYSTEMS, Boston, USA). cDNA was synthesized from each RNA sample (1 *μ*g) using the HiScript™ Q RT SuperMix for qPCR (+gDNA wiper) (R123-01; Vazyme, Nanjing, China). Real-time PCR assays were conducted with the primers for real-time PCR listed in Table S1 in Supplementary Material, available online at https://doi.org/10.1155/2017/8682057. Quantitative PCR was performed on samples deposited in triplicate using the standard curve mode protocol in which the calibration curve was generated using serial fivefold dilutions of 100 ng of total RNA. The RNA quantity was normalized using a probe specific for 16S rRNA.

### 2.4. RNA-Seq Analysis

To quantify the expression level of genes, HTSeq v0.6.1 was used to count the read numbers mapped to each gene [20]. Then, the FPKM (expected number of Fragments Per Kilobase of transcript sequence per Million base pairs sequenced) of each gene was calculated based on the length of the gene and the read counts mapped to this gene. Prior to differential gene expression analysis, for each sequenced library, the read counts were adjusted by edgeR program package through one scaling normalized factor [21]. In this study, we used the DEGSeq R package (1.20.0) to execute the differential expression analysis of two conditions [22]. Corrected *P* value of 0.005 and log2 (fold change) of 1 were set as the threshold for significantly differential expression. To analyze the gene structure of* R. anatipestifer *CH-1, Rockhopper was used to identify operons and transcription start sites. This program can be used for efficient and accurate analysis of bacterial RNA-Seq data and can aid in the elucidation of bacterial transcriptomes [23]. Moreover we used the aligned paired-end reads to infer the operonic structure of* R. anatipestifer* CH-1 transcripts. If genes obtained >20 reads aligning on both genes in a sequencing sample, they would be selected as potential in an operonic structure. Sequential genes that are present in operonic structure were merged together to form potential operonic transcripts (Table S2).

## 3. Results and Discussion

### 3.1. Growth of* R. anatipestifer* CH-1 in TSB and TSB with Dip

To evaluate the effect of iron restriction on the growth of* R. anatipestifer* CH-1, we grew* R. anatipestifer* CH-1 in TSB and TSB with 100 *μ*M Dip, which restricts most iron. Figure 1 showed that the growth of* R. anatipestifer* CH-1 was seriously hindered when iron was restricted, indicating that iron is an essential element for* R. anatipestifer*. Thus, this condition was suitable for performing RNA-Seq.

### 3.2. General Assessment of Iron Limitation Transcriptomic Datasets

**Figure 2:**
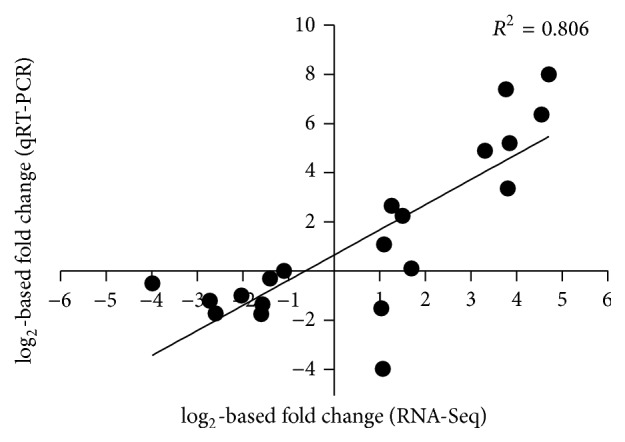
Validation of RNA-Seq data. Correlation analysis of log_2_-based fold changes between RNA-Seq data and qRT-PCR data for 20 genes of* R. anatipestifer* CH-1. The chart depicts a plot of RNA-Seq log_2_-based fold changes versus qRT-PCR log_2_-based fold changes for transcripts of genes in cultures of* R. anatipestifer *CH-1 grown in TSB+Dip medium versus TSB medium. A Pearson correlation coefficient of 0.806 was noted.

Over 95% of all clean reads aligned to coding regions of the* R. anatipestifer* CH-1 genome (Table 1). Since only one sample in the different iron condition was used to perform RNA-Seq, qRT-PCR validation was performed on the transcriptome data using a subset of 20 differentially regulated genes (Table S1). The transcriptome data generally corresponded well with the qRT-PCR data, with a Pearson correlation coefficient of 0.806 (Figure 2), illustrating that our RNA-Seq data were of suitable quality for transcriptome analysis.

**Figure 3:**
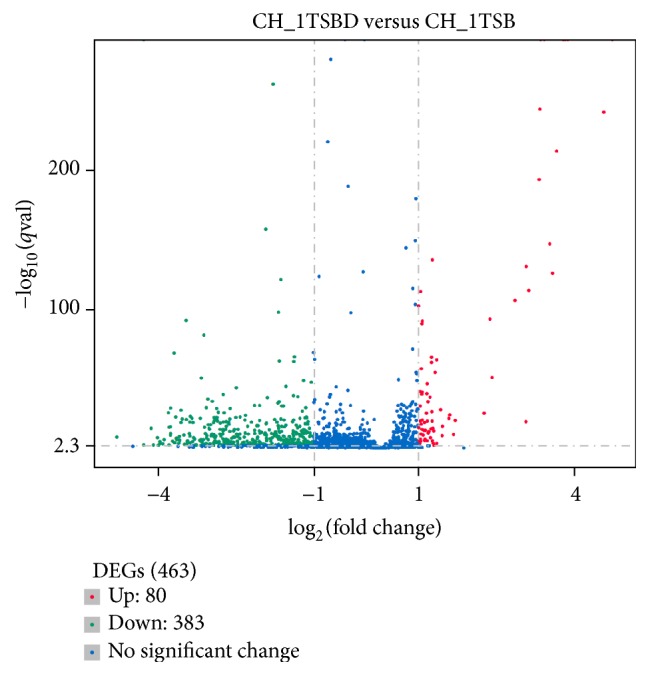
Differential gene transcription in cells grown in iron-limited TSB medium compared to TSB medium. The *x*-axis of the chart shows log2-based fold changes of transcripts in cells grown in iron-limited medium or TSB medium. The *y*-axis of the chart shows the statistical significance. Each dot in the chart represents one annotated gene. Red dots: upregulated, green dots: downregulated, and blue dots: no significant change.

Upon comparing cultures grown in TSB and TSB with Dip, overall differences in gene expression were observed (Figure 3). To examine these differences further, DEGs (differentially expressed genes) were identified using the DEseq package [22]. A total of 463 DEGs were identified, including 80 upregulated (Table 2) and 383 downregulated genes (Table S3). These genes represent 23% of the genome (2038 genes) [15]. The large number of DEGs suggests that iron-limited environments have global effects on* R. anatipestifer* CH-1. Since samples of different OD were used to perform RNA-Seq, this study can not exclude the fact that cell density might influence gene expression.

### 3.3. Genome-Wide Identification of* R. anatipestifer* CH-1 Genes in Operonic Structures

In addition to identifying gene boundaries, we drew on paired-end sequencing information to identify the* R. anatipestifer* CH-1 global operonic structure. In total, 377 genes were determined to be in operonic structures using this analysis, thus constituting 230 operons (Table S2). These genes represent 18% of the genome (2038 genes) [15].

### 3.4. Gene Ontology (GO) Annotation and Kyoto Encyclopedia of Genes and Genomes (KEGG) Pathway Mapping of DEGs

**Figure 4:**
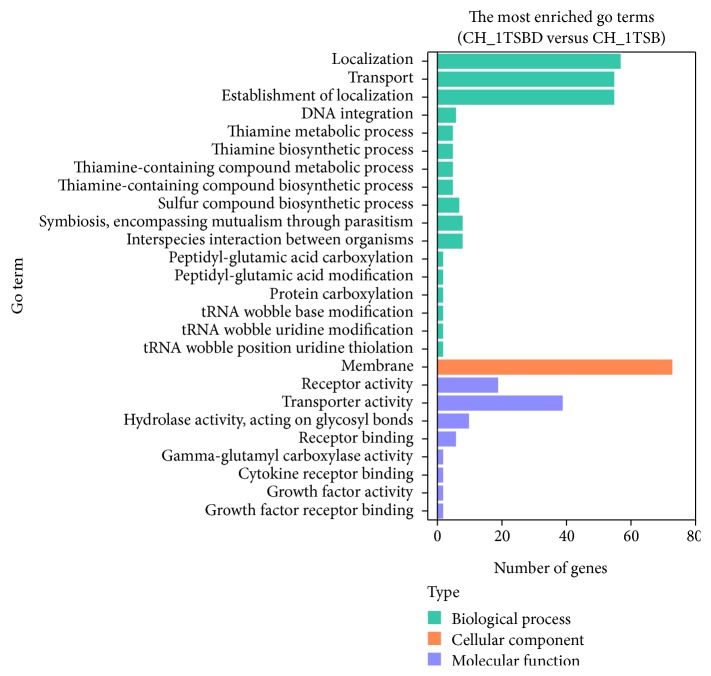
Role categories of genes from the transcriptome data. The numbers of genes that are up- and downregulated in* R. anatipestifer* CH-1 grown in iron-limited TSB medium versus TSB medium are categorized according to role categories. Some genes are listed in more than one category and so may be counted more than once.

The DEGs were assigned to 26 functional groups by enrichment analysis of Gene Ontology (GO) assignments [18]. In the three main GO categories of biological process, cellular component, and molecular function, genes in the role categories of “localization, transport, and establishment of localization” in biological process, “membrane” in cellular component or “receptor activity and transporter activity” in molecular function were notably up- or downregulated (Figure 4).

**Figure 5:**
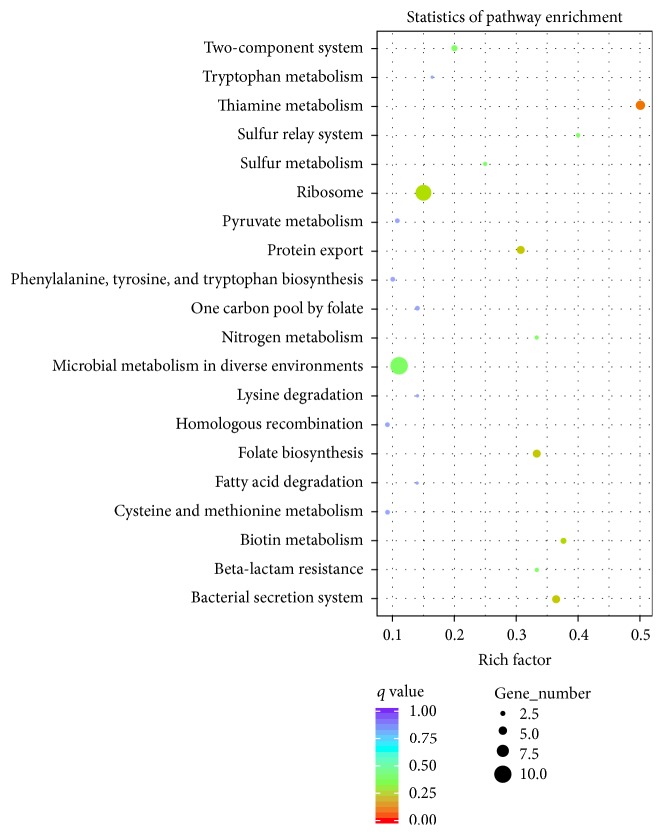
KEGG pathway enrichment analysis of differentially expressed genes between TSB and TSB with Dip. The *y*-axis of the chart shows the pathway name. The *x*-axis of the chart shows the Richness factor. The size of each point shows the number of genes in the pathway. The color of each point shows the* Q *value range.

The biological functions associated with the DEGs were further analyzed in terms of enriched Kyoto Encyclopedia of Genes and Genomes (KEGG) pathways [24], and a total of 20 pathways were predicted (Figure 5). Among these pathways, “microbial metabolism in diverse environments,” “ribosome,” and “thiamine metabolism” were the most highly represented categories (Figure 5).

### 3.5. Iron Limitation Increased the Transcription of Putative Iron Acquisition Systems

Genome sequence analysis indicated that* R. anatipestifer *CH-1 encodes Fe^2+^ and Fe^3+^ acquisition systems [15]. Once in the periplasm, Fe^2+^ is taken across the inner membrane via a divalent metal uptake system, such as the Feo system of* E. coli* [25] and the Yfe system of* Yersinia pestis* [26]. In this study, the predicted genes* feoB* (B739_0594) and* feoA* (B739_0595), which encode an Fe^2+^ transporter, were highly upregulated in the iron-limited condition, suggesting a role in the uptake of ferrous iron (Table 2). Sequence comparison revealed that all of the sequenced* R. anatipestifer *genomes have homologues of FeoA and FeoB of* R. anatipestifer* CH-1, with similarities of 100% for FeoA and between 88% and 100% for FeoB. In turn, these* R. anatipestifer* CH-1 genes have 33.45% and 32.89% identities to the FeoA and FeoB products of* E. coli*, respectively. In* E. coli, *this operon is regulated by Fur and is induced in acidic conditions [27]. The functions of FeoA and FeoB and their regulation in* R. anatipestifer *are underinvestigated.

In aerobic conditions, many bacteria produce siderophores to solubilize Fe^3+^. Then, siderophore-bound Fe^3+^ is taken up by TonB-dependent receptors [5]. Genome analysis revealed that there are at least 33 predicted TonB-dependent receptors in* R. anatipestifer *CH-1, some of which are predicted transporters for ferric-siderophore complexes or heme. In this study, 5 TonB-dependent receptors were upregulated (B739_0094, B739_0103, B739_0173, B739_1068, and B739_1416) in the presence of iron depletion. The expression levels of seven other putative TonB-dependent transporters (B739_0115, B739_0876, B739_1045, B739_1343, B739_0216, B739_0329, and B739_0389) (Table S3) were downregulated in the presence of iron depletion. Upregulated TonB-dependent receptors would be predicted to be involved in iron or hemin uptake, while the functions of all downregulated TonB-dependent receptors are presently unknown. Similar results have been obtained in other bacteria, such as* Pseudomonas fluorescens *[28].

TonB-dependent receptors rely on the accessory proteins ExbB, ExbD, and TonB for energy transduction. One TonB family protein and two sets of ExbB-ExbD-TonB were found and identified in* R. anatipestifer *[14]. In other bacteria, such as* E. coli *[29] and* Pseudomonas fluorescens *[28], the* tonB* gene is negatively regulated by iron. However, the transcription of* tonB *genes in* R. anatipestifer *CH-1 was not significantly changed in the iron-limited condition. To ensure the validity of the result, we also used qRT-PCR to measure* tonB* gene transcription in iron-limited conditions. The result was coincident with that of RNA-Seq. These results suggested that, in contrast to many bacteria, the* tonB* systems of* R. anatipestifer *CH-1 are not regulated by iron.

Once siderophore-bound Fe^3+^ is transported into the cytoplasm, the iron must be released from the siderophore. The first mechanism is that siderophore-bound Fe(III) is reduced to siderophore-bound Fe(II) followed by its spontaneous release due to the low affinity of iron Fe(II) with the siderophore. Another mechanism is that siderophore-bound Fe(III) is hydrolyzed by specialized enzymes, leading to a dramatic loss of complex stability and facilitating the subsequent removal of the iron [30] in a reduction process. In this study, a gene coding for a siderophore-interacting protein (B739_0608) was upregulated significantly in the presence of iron depletion. This siderophore-interacting protein is involved in iron acquisition and virulence in* R. anatipestifer* strain CH-3 [31]. Surprisingly, among the upregulated genes, we did not find any homologue gene related to siderophore synthesis.

### 3.6. A Putative Polysaccharide Utilization Locus of* R. anatipestifer *CH-1 Was Upregulated in Iron-Limited Conditions

In* Capnocytophaga canimorsus,* a member of the Bacteroidetes, a polysaccharide utilization system uses serotransferrin as an iron source [32]. Each polypeptide encoded by this locus is required for this iron uptake activity [32]. This type of system was named the iron capture system (ICS), and it contains seven genes:* icsA, icsC, icsD, icsE, icsF, icsG, *and* icsH *[32]. In this study, we identified a gene cluster (B739_0094, B739_0095, B739_0096, B739_0097, B739_0098, B739_0099, B739_0100, B739_0101, B739_0102, and B739_0103) (Table 2) involved in polysaccharide utilization, the expression of which was upregulated in the presence of iron depletion. Sequence comparison showed that the homologues of* icsC, icsD, icsE, icsF, icsG, *and* icsH* from* Capnocytophaga canimorsus* are B739_0103, B739_0102, B739_0101, B739_0100, B739_0099, and B739_0098, respectively, in the* R. anatipestifer *CH-1 genome. The homologue of* icsA, *B739_1068, was not cotranscribed with the others. Interestingly, some genes that were upregulated in the gene cluster, such as B739_0094, B739_0095, B739_0096, and B739_0097, were not predicted to contribute to the ICS system. Additionally, the* R. anatipestifer *CH-1 genome contains at least 6 polysaccharide utilization systems. In iron-limited conditions, three of the genes (locus B739_0094–B739_0103, locus B739_2091–B739_2093, and locus B739_0310–B739_0312) were upregulated (Table 2), while three other genes (locus B739_0115–B739_0118, locus B739_0875-B739_0876, and locus B739_1044-B739_1045) were downregulated (Table S3). Why some loci were upregulated and some loci were downregulated in the iron-limited condition is not currently understood.

### 3.7. Iron Limitation Increased Transcription of Putative Hemin Acquisition Systems

In the host, heme-containing proteins, such as hemoglobin, can be used as the main iron source by pathogenic bacteria [33]. Hemin uptake systems are regulated by iron in other bacteria [34, 35]. Here, putative genes involved in hemin uptake were more highly expressed in iron-limited cultures of* R. anatipestifer *CH-1 than in iron-replete cultures. Within the upregulated genes, the most highly expressed gene cluster was* FepA-hmuY* (B739_1416, B739_1417), which encodes a putative outer membrane ferrienterochelin, a colicin receptor and an HmuY-like hemophore protein. In* Porphyromonas gingivalis,* HmuY is a heme-binding lipoprotein associated with the outer membrane or secreted to the outside environment [36, 37]. Gene (B739_1415) adjacent to the* FepA-hmuY *operon was also upregulated in iron-limited medium. The functions of B739_1415, B739_1416, and B739_1417 in hemin utilization are underinvestigated in our group.

### 3.8. Transcription of Respiratory Chain Genes

In aerobic metabolism, the respiratory chain typically uses proteins that require iron as a cofactor [38]. When* R. anatipestifer *CH-1 was grown in iron-limited medium, the expression of genes coding for cytochrome biogenesis protein (B739_0948), periplasmic cytochrome c552 subunit (B739_0946), and cytochrome C (B739_0186) were downregulated (Table S3). It indicated that iron restriction hindered* R. anatipestifer* aerobic metabolism. Similarly, in other bacteria, such as* Pseudomonas fluorescens* Pf-5, the transcription levels of genes encoding cytochrome c-type biogenesis proteins (PFL_1684-88) and subunits of cbb3-type cytochrome c oxidases (PFL_1922-25, PFL_2834) are downregulated in iron-limited versus iron-replete medium [28].

### 3.9. Transcription of Genes Related to Natural Competence

Natural transformation refers to the process by which bacteria can actively take up and integrate exogenous DNA. Natural transformation is a major mechanism of horizontal gene transfer (HGT) and plays a prominent role in bacterial evolution [39]. The process of* Vibrio cholerae* natural transformation involves four steps: DNA-binding via type IV pili, DNA pulling via ComEA, DNA translocation via ComEC, and DNA recombination by the single-strand DNA-binding proteins DprA and RecA [40]. Previously, we found that* R. anatipestifer *CH-1 is naturally competent [41]. In* R. anatipestifer *CH-1, no putative type IV pilus locus is evident in the genome. In* R. anatipestifer *CH-1, two proteins that are predicted to be involved in the DNA uptake process, a ComEC homologue (B739_1095) and a gene encoding a single-strand DNA-binding protein (B739_1757), were downregulated in iron-limited conditions. One possibility for this phenomenon is that these proteins require iron for activity, as well as iron being predicted to be involved in the natural transformation process. This relationship between natural transformation and iron availability has not yet been described.

## 4. Conclusion

In this study, we examined the transcriptomic impact of iron limitation on* R. anatipestifer *CH-1 by comparing iron-limited TSB cultures with iron-replete TSB cultures. This transcriptome analysis identified numerous genes involved in* R. anatipestifer *CH-1 iron utilization. Under iron limitation, we observed changes in the transcription levels of genes related to iron homeostasis functions, such as the Feo system, the ICS system, and other iron uptake systems. Iron limitation also resulted in several unexpected responses, particularly the increased transcription of the ribosomal protein genes L18, L15, and L31. The data in this study were useful for identifying genes involved in iron utilization in* R. anatipestifer *CH-1 and for shedding light on the adaptation mechanisms of* R. anatipestifer *CH-1 in iron-limited environments, such as hosts.

## Supplementary Material

Table S1: Primer sequences for qRT-PCR validation of transcriptom data.
Table S2: The prediction of operons of *R. anatipestifer* CH-1 genes.
Table S3: Genes down-regulated in *Riemerellaanatipestifer* CH-1 in iron-depleted conditions.

## Figures and Tables

**Table 1:** Summary of Illumina RNA-Seq data.

Sample	Total reads	Total mapped	Clean data^*∗*^ (Gb)	Percentage of sequence reads mapped
CH_1TSB	17948156	17106745	2.24	95.31%
CH_1TSBD	21489778	21099289	2.68	98.18%

^*∗*^Clean data were obtained from raw data by removing reads containing adapter and poly-N and low-quality reads.

**Table 2:** Genes upregulated in *Riemerella anatipestifer* CH-1 in iron-depleted conditions.

Gene ID	Gene name	log2.Fold_change.	*P* value	Description
13715178	B739_0074	1.289	0.00021641	Hypothetical protein
13715197	B739_0093	2.8509	2.72*E* − 108	Hypothetical protein
13715198	B739_0094	1.2615	8.08*E* − 28	Outer membrane receptor for Fe3+FecA
13715199	B739_0095	1.5902	5.31*E* − 23	Type I deoxyribonuclease HsdR
13715200	B739_0096	3.3265	4.50*E* − 246	Hypothetical protein
13715201	B739_0097	3.1172	1.67*E* − 115	Carbohydrate-binding protein
13715202	B739_0098	3.3417	0	Hypothetical protein
13715203	B739_0099	3.5712	5.25*E* − 128	Hypothetical protein
13715204	B739_0100	3.5135	5.06*E* − 149	Hypothetical protein
13715205	B739_0101	3.0646	1.23*E* − 132	Substrate import-associated zinc metallohydrolase
13715206	B739_0102	3.4075	0	Glycan metabolism protein RagB
13715207	B739_0103	3.8099	0	TonB-linked outer membrane protein, SusC/RagA family
13715231	B739_0127	1.275	1.56*E* − 06	DNA-binding protein
13715277	B739_0173	3.3109	2.55*E* − 195	TonB-dependent receptor CirA, mostly Fe transport
13715278	B739_0174	3.6434	5.92*E* − 216	Hypothetical protein
13715279	B739_0175	2.413	5.96*E* − 53	Ankyrin
13715280	B739_0176	3.0584	1.11*E* − 20	Predicted periplasmic protein
13715281	B739_0177	1.0467	3.85*E* − 06	Nitric oxide synthase
13715373	B739_2137	1.1196	6.02*E* − 07	Camphor resistance protein CrcB; integral membrane protein possibly involved in chromosome condensation [cell division and chromosome partitioning]
13715432	B739_0891	1.0093	1.05*E* − 06	Hypothetical protein
13715452	B739_0912	1.0968	6.65*E* − 11	Ribonuclease III
13715453	B739_0913	1.0147	0.0019348	Hypothetical protein
13715513	B739_0973	1.0814	1.74*E* − 42	50S ribosomal protein L16/L10E
13715515	B739_0975	1.0712	4.92*E* − 15	30S ribosomal protein S17
13715522	B739_0982	1.1625	3.94*E* − 41	50S ribosomal protein L18
13715525	B739_0985	1.2524	1.07*E* − 63	50S ribosomal protein L15
13715526	B739_0986	1.0086	2.96*E* − 104	Preprotein translocase subunit SecY
13715542	−//−	1.4285	1.38*E* − 29	tRNA-Glu
13715606	B739_1068	3.8593	0	FecA
13715627	B739_1089	1.3526	1.76*E* − 65	Hypothetical protein
13715649	B739_1112	1.0673	5.05*E* − 41	50S ribosomal protein L31
13715783	B739_1246	1.0718	1.24*E* − 91	30S ribosomal protein S16
13715836	B739_1299	1.2361	5.75*E* − 09	Hypothetical protein
13715897	B739_1360	1.3576	0.0002407	Hypothetical protein
13715932	B739_1395	1.4655	1.89*E* − 17	2-Amino-4-hydroxy-6-hydroxymethyldihydropteridine pyrophosphokinase
13715934	B739_1397	1.08	1.36*E* − 93	Outer membrane protein-related peptidoglycan-associated (lipo)protein
13715952	B739_1415	4.7103	0	Hypothetical protein
13715953	B739_1416	3.7691	0	FepA
13715954	B739_1417	4.5479	5.15*E* − 244	HmuY
13715956	B739_1419	1.2458	2.43*E* − 15	Restriction endonuclease S subunits, Hsds
13716000	B739_1467	1.605	7.16*E* − 26	Hypothetical protein
13716023	B739_1491	1.2534	2.55*E* − 67	Hypothetical protein
13716028	B739_1496	1.0255	0.00022217	Hypothetical protein
13716038	B739_1506	1.2051	1.41*E* − 13	Phosphate transport regulator
13716056	B739_1525	1.0126	2.76*E* − 16	OmpA
13716068	B739_1537	1.1923	2.42*E* − 35	Thioredoxin
13716179	B739_1648	1.1687	1.56*E* − 06	Hypothetical protein
13716365	B739_1842	1.4584	1.63*E* − 10	Hypothetical protein
13716406	B739_1883	1.1398	2.85*E* − 14	Preprotein translocase subunit SecG
13716420	B739_1898	1.1221	8.61*E* − 20	3-Oxoacyl-(acyl-carrier-protein) synthase III
13716438	B739_1916	1.1349	1.80*E* − 07	Hypothetical protein
13716459	B739_1938	1.0469	7.76*E* − 115	Hypothetical protein
13716524	B739_2003	1.1565	1.21*E* − 26	Polyisoprenoid-binding protein; YceI-like domain
13716533	B739_2012	1.3031	0.0098864	Prevent-host-death protein; Antitoxin Phd_YefM, type II toxin-antitoxin system
13716610	B739_2089	1.0236	0.00048509	Porin
13716613	B739_2092	1.0406	3.79*E* − 32	Starch binding outer membrane protein SusD
13716712	B739_0221	1.0457	3.80*E* − 21	Gliding motility protein GldL
13716745	B739_0254	1.1712	1.78*E* − 48	Hypothetical protein
13716748	B739_0257	1.0548	1.69*E* − 11	Hypothetical protein
13716800	B739_0310	1.1729	4.55*E* − 21	Carbohydrate-binding protein SusD
13716803	B739_0313	1.0601	2.33*E* − 59	Ribonuclease G
13716804	B739_0314	1.3277	1.60*E* − 56	Bacterial nucleoid DNA-binding protein
13716824	B739_0335	1.2846	9.72*E* − 16	Hypothetical protein
13716825	B739_0336	1.0435	1.66*E* − 07	Hypothetical protein
13716826	B739_0337	1.0406	3.10*E* − 06	Ras_like_GTPase
13716848	B739_0360	1.2725	1.12*E* − 137	50S ribosomal protein L11
13716849	B739_0361	1.0413	1.23*E* − 42	Transcription antiterminator
13716885	B739_0397	1.0567	6.51*E* − 19	IMP dehydrogenase/GMP reductase
13716908	B739_0420	1.0126	2.35*E* − 05	Sec-independent protein secretion pathway component
13716924	B739_0436	1.019	0.00033736	Predicted glycosyltransferases
13716964	B739_0476	1.2682	6.15*E* − 21	Hypothetical protein
13716978	B739_0490	2.3711	4.94*E* − 95	Ferritin-like domain
13717035	B739_0547	1.2256	9.53*E* − 39	RNA polymerase Rpb6
13717082	B739_0594	1.082	1.16*E* − 21	Iron transporter FeoB
13717083	B739_0595	2.259	5.94*E* − 27	Iron transporter FeoA
13717096	B739_0608	1.6768	3.57*E* − 11	Oxidoreductase; siderophore-interacting protein [inorganic ion transport and metabolism]ViuB
13717113	B739_0625	1.7097	2.66*E* − 21	RNA polymerase sigma factor
13717239	B739_0753	1.1957	3.41*E* − 12	Transthyretin-like protein
13717245	B739_0759	1.1112	2.64*E* − 14	Iron-sulfur binding protein

## Abbreviations

RNA-Seq: RNA sequencing
qRT-PCR: Quantitative reverse transcription polymerase chain reaction
HGT: Horizontal gene transfer
DEGs: Differentially expressed genes
KEGG: Kyoto encyclopedia of genes and genomes
GO: Gene Ontology
ICS: Iron capture system
FPKM: Expected number of Fragments Per Kilobase of transcript sequence per Million base pairs sequenced.
