# Supplementary material for: Identifying the Genes Responsible for Iron-Limited Condition in Riemerella anatipestifer CH-1 through RNA-Seq-Based Analysis
Source: Biomed Res Int. 2017 Apr 30;2017:8682057. doi: 10.1155/2017/8682057 (PMC5429918; doi:10.1155/2017/8682057)
Supplement: Supplementary file 1 — Table S1: Primer sequences for qRT-PCR validation of transcriptom data. Table S2: The prediction of operons of R. anatipestifer CH-1 genes. Table S3: Genes down-regulated in Riemerellaanatipestifer CH-1 in iron-depleted conditions. [file 8682057.f1.pdf]

TableS1. Primer sequences for qRT-PCR validation of transcriptom data

Gene ID	Annotated functions	Primer names	Primer sequences	Product sizes (bp)	log2.Fold_ change
B739_0094	Outer membrane receptor for Fe3+ FecA	0094P1	TGGAACCTTTGGAATGTGTC	130	2.65
		0094P2	AATCCACATCTGCAACTCTG		
B739_0103	TonB-linked outer membrane protein, SusC/RagA family	0103P1	TAATACCAGCCGTACATTGC	153	3.359
		0103P2	TATTCAAGTTCCGTCTGCAC		
B739_0173	TonB-denpendent receptor CirA, mostly Fe transport	0173P1	AGTACCTGCATCTACCTACG	114	4.89
		0173P2	GCATCATCAGCGATACTTCC		
B739_0975	30S ribosomal protein S17	0975P1	GAATCGGAGTAGTTTCGAGC	153	-3.98
		0975P2	CAGTATCGCCTTCGTTACAC		
B739_1068	FecA	1068P1	AGCTGGAACTACGAACTAGG	139	5.21
		1068P2	ATAAATCGCCTTGTCCTGTG		
B739_1415	Hypothetical protein	1415P1	ACATTGGAGACAAGATTGGC	173	7.998
		1415P2	AGTGTATCCAGGTTCCAGAG		
B739_1416	FepA	1416P1	GCTAGTGATTACCCACAACC	121	7.4
		1416P2	TAAATCCAAAGTTCCTGCCG		
B739_1417	HmuY	1417P1	ACCACAGAGTGGGATATAGG	122	6.37
		1417P2	GGAGCTTCCTTTACATCTGC		
B739_2092	Starch binding outer membrane protein SusD	2092P1	AAGAGCACTGAGTTATGCAC	194	-1.51
		2092P2	TATCTGCTCTCCCGTATTCG		
B739_0594	Iron transporter FeoB	0594P1	TTGGTAACGCCGTTTATGAC	123	1.1
		0594P2	CATACCCATAAGTGCCATCG		
B739_0595	Iron transporter FeoA	0595P1	GATAGCCAGCTAGAAATGCC	163	1.5
		0595P2	GAATATAAGCCGCCTCTTCC		
B739_0625	RNA polymerase sigma factor	0625P1	CCATATTAGCGAACCTACGG	172	0.11
		0625P2	GCTACAATCCGTATGCTCTG		
B739_0071	Outer membrane receptor proteins, mostly Fe transport	0071P1	AAGTGATTGCTCATCCTTCG	142	-1.73
		0071P2	CTCGGAAACTTACCCAATCC		
B739_0876	TonB-linked outer membrane protein, SusC/RagA family	0876P1	CATCAGCCTCTTTCAGTTGG	188	-1.21
		0876P2	GTCTGTGTTTGGAGCTACTG		
B739_1045	TonB-linked outer membrane protein, SusC/RagA family	1045P1	AACCACCAGTCCTAATTCAG	141	-1
		1045P2	CTGCCGAATACACTAGAACC		
B739_1343	Outer membrane receptor proteins, mostly Fe transport [Inorganic ion transport and metabolism];CirA	1343P1	TGGGAAACAGGTCTATCAGG	165	0
		1343P2	CATATCGTACCGCAAAGCTG		
B739_1968	RNA polymerase sigma factor;	1968P1	TTACATCACTTCCGACATGC	198	-0.5
		1968P2	TCGCCCTCTTCTAAATTAGC		
B739_0329	Outer membrane receptor proteins, mostly Fe transport	0329P1	TCAGAACGCCTTAATGACTG	118	-0.3
		0329P2	TGATAAGCCCTGAAAGTACC		
B739_0406	Outer membrane receptor proteins, mostly Fe transport	0406P1	GCTAATGGAAACCTTTGGAC	155	-1.75
		0406P2	CCCTGACGAGTTACTTTCTG		
B739_0782	Hemolysin D	0782P1	ATGCCACAAGTCTATGAAGC	152	-1.35
		0782P2	CTAGCCGCATCTAGTTTAGC		

Table S2. The prediction of operons of *R. anatipestifer* CH-1 genes.

Start	Stop	Strand	Number of Genes	Genes
1105	2735	-	2	B739_0001, B739_0002
4847	6325	-	2	B739_0005, B739_0006
6509	7187	-	2	B739_0007, B739_0008
7423	8690	-	2	B739_0009, B739_0010
9242	11906	-	3	B739_0011, B739_0012, B739_0013
12233	12924	-	2	B739_0014, B739_0015
15891	18950	-	4	B739_0018, B739_0019, B739_0020, B739_0021
21422	22311	-	3	B739_0026, B739_0027, B739_0028
40060	58740	-	11	B739_0042, B739_0043, B739_0044, B739_0045, B739_0046, B739_0047, B739_0048, B739_0049, B739_0050, B739_0051, B739_0052
59502	63179	-	5	B739_0054, B739_0055, B739_0056, B739_0057, B739_0058
64516	69207	-	4	B739_0060, B739_0061, B739_0062, B739_0063
74575	76462	-	2	B739_0069, B739_0070
80021	81275	+	2	B739_0072, B739_0073
81320	89466	-	9	B739_0074, B739_0075, B739_0076, B739_0077, B739_0078, B739_0079, B739_0080, B739_0081, B739_0082
89766	92021	+	3	B739_0083, B739_0084, B739_0085
92137	96173	+	3	B739_0086, B739_0087, B739_0088
96498	98715	+	3	B739_0089, B739_0090, B739_0091
101060	114851	-	10	B739_0094, B739_0095, B739_0096, B739_0097, B739_0098, B739_0099, B739_0100, B739_0101, B739_0102, B739_0103
114998	115907	-	2	B739_0104, B739_0105
120221	122895	+	2	B739_0109, B739_0110
128078	135126	+	4	B739_0115, B739_0116, B739_0117, B739_0118
135208	139563	-	2	B739_0119, B739_0120
140311	143032	-	2	B739_0122, B739_0123
143925	144277	+	2	B739_0125, B739_0126
146287	147040	+	2	B739_0130, B739_0131
147184	149079	-	2	B739_0132, B739_0133
150391	150863	-	2	B739_0135, B739_0136
151202	153555	-	4	B739_0137, B739_0138, B739_0139, B739_0140
155702	160314	-	3	B739_0143, B739_0144, B739_0145
160425	161656	-	2	B739_0146, B739_0147
162544	165035	+	3	B739_0149, B739_0150, B739_0151
167828	169715	-	2	B739_0155, B739_0156
170756	173856	-	4	B739_0158, B739_0159, B739_0160, B739_0161
173970	175924	-	3	B739_0162, B739_0163, B739_0164
176007	178651	-	4	B739_0165, B739_0166, B739_0167, B739_0168
178864	180891	-	2	B739_0169, B739_0170

181056	183166	-	2	B739_0171, B739_0172
183371	192169	+	6	B739_0173, B739_0174, B739_0175, B739_0176, B739_0177, B739_0178
193007	194258	+	2	B739_0180, B739_0181
194413	196270	+	2	B739_0182, B739_0183
196255	197020	-	2	B739_0184, B739_0185
197154	200304	+	3	B739_0186, B739_0187, B739_0188
200427	206153	+	7	B739_0189, B739_0190, B739_0191, B739_0192, B739_0193, B739_0194, B739_0195
207529	208828	-	2	B739_0197, B739_0198
211570	216944	-	4	B739_0201, B739_0202, B739_0203, B739_0204
219254	220719	-	3	B739_0208, B739_0209, B739_0210
224303	226748	-	3	B739_0213, B739_0214, B739_0215
227011	230818	+	2	B739_0216, B739_0217
232997	236734	-	3	B739_0220, B739_0221, B739_0222
236914	241042	-	3	B739_0223, B739_0224, B739_0225
241191	245789	+	5	B739_0226, B739_0227, B739_0228, B739_0229, B739_0230
245835	249104	-	3	B739_0231, B739_0232, B739_0233
249167	251009	+	3	B739_0234, B739_0235, B739_0236
251160	252944	-	2	B739_0237, B739_0238
253793	255687	-	3	B739_0239, B739_0240, B739_0241
260360	261615	-	2	B739_0247, B739_0248
262681	268123	-	4	B739_0250, B739_0251, B739_0252, B739_0253
268572	270717	+	3	B739_0255, B739_0256, B739_0257
270785	272531	-	2	B739_0258, B739_0259
283363	292172	+	5	B739_0266, B739_0267, B739_0268, B739_0269, B739_0270
299166	300376	-	2	B739_0280, B739_0281
302476	304336	-	2	B739_0286, B739_0287
308283	309731	-	2	B739_0292, B739_0293
309994	313701	+	3	B739_0294, B739_0295, B739_0296
313794	317184	+	4	B739_0297, B739_0298, B739_0299, B739_0300
318545	320270	-	2	B739_0302, B739_0303
329990	337015	-	3	B739_0310, B739_0311, B739_0312
339265	341856	+	3	B739_0315, B739_0316, B739_0317
350584	353054	+	2	B739_0325, B739_0326
353216	356136	+	2	B739_0327, B739_0328
366429	369090	-	6	B739_0333, B739_0334, B739_0335, B739_0336, B739_0337, B739_0338
369564	370326	-	2	B739_0339, B739_0340
370412	373512	-	5	B739_0341, B739_0342, B739_0343, B739_0344, B739_0345
374414	376918	-	2	B739_0347, B739_0348
379646	387824	-	2	B739_0351, B739_0352
389168	391756	+	2	B739_0355, B739_0356

391828	393934	-	4	B739_0357, B739_0358, B739_0359, B739_0360
394162	394858	-	2	B739_0361, B739_0362
398137	399329	-	2	B739_0365, B739_0366
399795	401734	+	2	B739_0368, B739_0369
401736	404308	-	4	B739_0370, B739_0371, B739_0372, B739_0373
406940	413140	+	7	B739_0375, B739_0376, B739_0377, B739_0378, B739_0379, B739_0380, B739_0381
414663	417114	+	3	B739_0383, B739_0384, B739_0385
417313	418158	+	2	B739_0386, B739_0387
419260	423140	+	2	B739_0389, B739_0390
423362	424742	+	2	B739_0391, B739_0392
428357	434168	+	5	B739_0396, B739_0397, B739_0398, B739_0399, B739_0400
434256	437424	-	2	B739_0401, B739_0402
437602	439468	-	2	B739_0403, B739_0404
444059	446739	+	3	B739_0409, B739_0410, B739_0411
446747	451623	-	5	B739_0412, B739_0413, B739_0414, B739_0415, B739_0416
451784	452792	-	2	B739_0417, B739_0418
452857	453690	-	2	B739_0419, B739_0420
453800	461669	-	8	B739_0421, B739_0422, B739_0423, B739_0424, B739_0425, B739_0426, B739_0427, B739_0428
461716	462639	+	2	B739_0429, B739_0430
462627	467642	-	6	B739_0431, B739_0432, B739_0433, B739_0434, B739_0435, B739_0436
470207	472360	+	2	B739_0438, B739_0439
472416	476313	-	6	B739_0440, B739_0441, B739_0442, B739_0443, B739_0444, B739_0445
478625	481141	-	2	B739_0448, B739_0449
482819	487215	+	6	B739_0451, B739_0452, B739_0453, B739_0454, B739_0455, B739_0456
487379	494390	+	6	B739_0457, B739_0458, B739_0459, B739_0460, B739_0461, B739_0462
494851	497527	+	2	B739_0464, B739_0465
497550	500169	-	2	B739_0466, B739_0467
500895	502827	+	2	B739_0469, B739_0470
507067	508018	-	2	B739_0475, B739_0476
508083	508497	-	2	B739_0477, B739_0478
510474	513811	-	4	B739_0480, B739_0481, B739_0482, B739_0483
513979	517905	-	4	B739_0484, B739_0485, B739_0486, B739_0487
519813	523304	+	3	B739_0491, B739_0492, B739_0493
527140	529344	-	2	B739_0497, B739_0498
532119	533787	+	2	B739_0501, B739_0502
539555	541267	-	3	B739_0507, B739_0508, B739_0509
542269	546165	+	4	B739_0511, B739_0512, B739_0513, B739_0514
547408	548632	-	2	B739_0516, B739_0517
549924	551473	+	2	B739_0519, B739_0520

551868	554373	-	2	B739_0522, B739_0523
554475	561737	-	7	B739_0524, B739_0525, B739_0526, B739_0527, B739_0528, B739_0529, B739_0530
561797	564199	-	3	B739_0531, B739_0532, B739_0533
567100	567704	+	2	B739_0536, B739_0537
567867	569951	+	2	B739_0538, B739_0539
570582	573541	+	2	B739_0541, B739_0542
579725	582889	+	4	B739_0548, B739_0549, B739_0550, B739_0551
583661	585350	+	3	B739_0553, B739_0554, B739_0555
585364	587203	-	2	B739_0556, B739_0557
587333	590761	-	3	B739_0558, B739_0559, B739_0560
593696	595261	+	2	B739_0563, B739_0564
596359	598042	+	2	B739_0566, B739_0567
604311	607733	+	4	B739_0573, B739_0574, B739_0575, B739_0576
607863	608479	+	2	B739_0577, B739_0578
608564	619463	-	6	B739_0579, B739_0580, B739_0581, B739_0582, B739_0583, B739_0584
629647	630783	+	2	B739_0592, B739_0593
631055	633379	-	2	B739_0594, B739_0595
634159	636583	+	2	B739_0597, B739_0598
652464	653070	+	2	B739_0609, B739_0610
653996	657347	+	2	B739_0612, B739_0613
658706	661205	-	3	B739_0616, B739_0617, B739_0618
661669	664678	-	4	B739_0619, B739_0620, B739_0621, B739_0622
664776	666259	-	3	B739_0623, B739_0624, B739_0625
668010	670377	-	3	B739_0627, B739_0628, B739_0629
670433	674171	-	3	B739_0630, B739_0631, B739_0632
674663	677830	-	3	B739_0633, B739_0634, B739_0635
685072	687429	-	2	B739_0643, B739_0644
689504	691762	-	3	B739_0647, B739_0648, B739_0649
691959	692826	+	3	B739_0650, B739_0651, B739_0652
694618	697591	-	2	B739_0654, B739_0655
697679	700971	-	2	B739_0656, B739_0657
701075	707921	-	5	B739_0658, B739_0659, B739_0660, B739_0661, B739_0662
709460	710025	-	3	B739_0665, B739_0666, B739_0667
710333	715040	-	4	B739_0668, B739_0669, B739_0670, B739_0671
715711	719469	-	5	B739_0673, B739_0674, B739_0675, B739_0676, B739_0677
719555	727927	-	6	B739_0678, B739_0679, B739_0680, B739_0681, B739_0682, B739_0683
728143	728788	+	2	B739_0684, B739_0685
729636	731112	-	3	B739_0687, B739_0688, B739_0689
733070	737187	-	3	B739_0692, B739_0693, B739_0694
737825	739542	+	3	B739_0696, B739_0697, B739_0698

744540	750396	-	5	B739_0703, B739_0704, B739_0705, B739_0706, B739_0707
751530	755588	+	4	B739_0709, B739_0710, B739_0711, B739_0712
755667	756824	-	2	B739_0713, B739_0714
756949	759435	+	2	B739_0715, B739_0716
759651	761977	-	3	B739_0717, B739_0718, B739_0719
762041	765079	+	2	B739_0720, B739_0721
765082	769400	-	3	B739_0722, B739_0723, B739_0724
769581	771625	+	3	B739_0725, B739_0726, B739_0727
774122	777067	-	4	B739_0732, B739_0733, B739_0734, B739_0735
778300	779217	+	2	B739_0737, B739_0738
779330	783261	-	5	B739_0739, B739_0740, B739_0741, B739_0742, B739_0743
783420	785635	-	2	B739_0744, B739_0745
786319	788925	+	2	B739_0747, B739_0748
790887	796188	-	7	B739_0751, B739_0752, B739_0753, B739_0754, B739_0755, B739_0756, B739_0757
796295	797097	-	2	B739_0758, B739_0759
799170	800460	-	2	B739_0761, B739_0762
800544	804349	-	2	B739_0763, B739_0764
805965	811034	+	3	B739_0766, B739_0767, B739_0768
813844	814259	+	2	B739_0771, B739_0772
814317	815871	-	2	B739_0773, B739_0774
815916	818005	+	3	B739_0775, B739_0776, B739_0777
820886	826573	-	3	B739_0780, B739_0781, B739_0782
829985	832589	+	2	B739_0786, B739_0787
838954	843383	-	2	B739_0793, B739_0794
844691	846455	-	2	B739_0796, B739_0797
852194	853191	+	2	B739_0801, B739_0802
853202	858722	-	3	B739_0803, B739_0804, B739_0805
858889	861987	+	3	B739_0806, B739_0807, B739_0808
862291	865957	-	2	B739_0809, B739_0810
866324	869868	+	3	B739_0811, B739_0812, B739_0813
872930	877360	+	4	B739_0817, B739_0818, B739_0819, B739_0820
878211	880723	-	3	B739_0821, B739_0822, B739_0823
882638	884813	+	3	B739_0825, B739_0826, B739_0827
885012	890334	+	5	B739_0828, B739_0829, B739_0830, B739_0831, B739_0832
890397	892429	+	3	B739_0833, B739_0834, B739_0835
901514	902600	-	2	B739_0839, B739_0840
903650	915289	+	10	B739_0842, B739_0843, B739_0844, B739_0845, B739_0846, B739_0847, B739_0848, B739_0849, B739_0850, B739_0851
915371	919286	+	3	B739_0852, B739_0853, B739_0854
919371	921559	-	2	B739_0855, B739_0856

923512	925750	-	2	B739_0858, B739_0859
929289	932988	+	4	B739_0864, B739_0865, B739_0866, B739_0867
934662	940893	+	4	B739_0870, B739_0871, B739_0872, B739_0873
940958	946265	-	3	B739_0874, B739_0875, B739_0876
952536	954598	-	2	B739_0882, B739_0883
956511	957885	+	3	B739_0886, B739_0887, B739_0888
959445	964502	+	5	B739_0890, B739_0891, B739_0892, B739_0893, B739_0894
964510	965796	-	2	B739_0895, B739_0896
968175	970465	+	2	B739_0898, B739_0899
971106	972411	+	2	B739_0901, B739_0902
973628	974856	-	3	B739_0904, B739_0905, B739_0906
977804	981958	+	5	B739_0910, B739_0911, B739_0912, B739_0913, B739_0914
984759	988125	-	2	B739_0917, B739_0918
990618	992345	+	2	B739_0922, B739_0923
992309	994363	-	3	B739_0924, B739_0925, B739_0926
1001275	1004390	+	5	B739_0931, B739_0932, B739_0933, B739_0934, B739_0935
1004478	1006944	+	2	B739_0936, B739_0937
1007528	1009332	-	2	B739_0939, B739_0940
1012308	1013534	-	2	B739_0943, B739_0944
1013857	1018052	+	4	B739_0945, B739_0946, B739_0947, B739_0948
1018114	1021918	-	5	B739_0949, B739_0950, B739_0951, B739_0952, B739_0953
1024894	1026886	+	2	B739_0955, B739_0956
1026998	1031015	+	4	B739_0957, B739_0958, B739_0959, B739_0960
1031875	1034980	-	2	B739_0962, B739_0963
1037161	1038424	-	2	B739_0965, B739_0966
1038727	1041105	+	4	B739_0967, B739_0968, B739_0969, B739_0970
1041392	1042499	+	2	B739_0971, B739_0972
1042610	1045046	+	7	B739_0973, B739_0974, B739_0975, B739_0976, B739_0977, B739_0978, B739_0979
1045233	1052473	+	14	B739_0980, B739_0981, B739_0982, B739_0983, B739_0984, B739_0985, B739_0986, B739_0987, B739_0988, B739_0989, B739_0990, B739_0991, B739_0992, B739_0993
1052562	1054648	-	2	B739_0994, B739_0995
1057608	1061130	-	3	B739_0998, B739_0999, B739_1000
1063152	1066771	-	4	B739_1002, B739_1003, B739_1004, B739_1005
1066880	1071179	-	2	B739_1006, B739_1007
1071324	1072884	+	2	B739_1008, B739_1515
1072945	1074555	+	2	B739_1010, B739_1011
1074634	1078884	+	4	B739_1012, B739_1013, B739_1014, B739_1015
1078990	1081073	+	2	B739_1016, B739_1017
1083101	1086822	+	3	B739_1021, B739_1022, B739_1023

1087668	1090261	+	2	B739_1025, B739_1026
1092104	1094300	-	2	B739_1028, B739_1029
1099681	1102255	+	2	B739_1035, B739_1036
1102313	1104390	-	3	B739_1037, B739_1038, B739_1039
1105286	1112898	-	2	B739_1042, B739_1043
1113069	1117482	-	2	B739_1044, B739_1045
1117860	1120371	+	4	B739_1046, B739_1047, B739_1048, B739_1049
1124489	1127169	+	2	B739_1055, B739_1056
1127281	1129283	+	2	B739_1057, B739_1058
1135173	1137530	+	2	B739_1060, B739_1061
1138466	1141985	+	3	B739_1063, B739_1064, B739_1065
1147558	1148464	+	2	B739_1070, B739_1071
1149888	1151263	+	2	B739_1073, B739_1074
1151325	1152270	-	2	B739_1075, B739_1076
1153538	1155482	-	3	B739_1078, B739_1079, B739_1080
1155641	1157028	+	2	B739_1081, B739_1082
1158521	1163978	+	3	B739_1085, B739_1086, B739_1087
1165702	1169899	-	2	B739_1089, B739_1090
1174248	1176411	+	2	B739_1094, B739_1095
1176536	1180073	+	3	B739_1096, B739_1097, B739_1098
1182514	1186004	+	2	B739_1101, B739_1102
1186825	1193178	+	3	B739_1104, B739_1105, B739_1106
1194826	1195786	+	2	B739_1108, B739_1109
1197974	1199479	-	2	B739_1111, B739_1112
1199648	1203423	+	4	B739_1113, B739_1114, B739_1115, B739_1116
1204487	1207624	-	3	B739_1118, B739_1119, B739_1120
1211771	1218459	+	5	B739_1124, B739_1125, B739_1126, B739_1127, B739_1128
1219634	1226489	+	7	B739_1130, B739_1131, B739_1132, B739_1133, B739_1134, B739_1135, B739_1136
1226645	1229762	+	4	B739_1137, B739_1138, B739_1139, B739_1140
1231768	1233549	+	3	B739_1143, B739_1144, B739_1145
1233654	1236806	+	2	B739_1146, B739_1147
1238871	1240185	+	2	B739_1150, B739_1151
1240309	1241272	+	2	B739_1152, B739_1153
1241355	1243108	+	2	B739_1154, B739_1155
1243166	1245046	+	2	B739_1156, B739_1157
1245154	1247241	+	2	B739_1158, B739_1159
1247357	1253470	+	6	B739_1160, B739_1161, B739_1162, B739_1163, B739_1164, B739_1165
1253598	1255446	+	3	B739_1166, B739_1167, B739_1168
1262371	1265441	+	3	B739_1173, B739_1174, B739_1175
1266283	1267735	+	2	B739_1177, B739_1178

1269525	1271994	-	3	B739_1180, B739_1181, B739_1182
1278150	1283338	-	4	B739_1187, B739_1188, B739_1189, B739_1190
1284444	1292533	+	9	B739_1193, B739_1194, B739_1195, B739_1196, B739_1197, B739_1198, B739_1199, B739_1200, B739_1201
1295031	1297729	-	2	B739_1205, B739_1206
1297887	1300387	+	2	B739_1207, B739_1208
1301926	1303643	+	2	B739_1210, B739_1211
1303755	1305965	+	2	B739_1212, B739_1213
1308310	1310037	-	2	B739_1216, B739_1217
1311409	1313245	+	2	B739_1219, B739_1220
1313370	1315421	+	2	B739_1221, B739_1222
1316479	1322824	-	6	B739_1224, B739_1225, B739_1226, B739_1227, B739_1228, B739_1229
1323034	1324975	+	2	B739_1230, B739_1231
1325004	1330670	-	6	B739_1232, B739_1233, B739_1234, B739_1235, B739_1236, B739_1237
1330945	1332111	+	2	B739_1238, B739_1239
1332150	1336334	-	5	B739_1240, B739_1241, B739_1242, B739_1243, B739_1244
1336483	1337558	-	2	B739_1245, B739_1246
1340035	1342889	-	2	B739_1250, B739_1251
1343005	1345037	-	2	B739_1252, B739_1253
1348333	1353405	+	2	B739_1256, B739_1257
1353476	1354295	-	2	B739_1258, B739_1259
1354820	1359570	+	6	B739_1261, B739_1262, B739_1263, B739_1264, B739_1265, B739_1266
1359852	1362734	+	9	B739_1267, B739_1268, B739_1269, B739_1270, B739_1271, B739_1272, B739_1273, B739_1274, B739_1275
1362861	1377988	+	20	B739_1276, B739_1277, B739_1278, B739_1279, B739_1280, B739_1281, B739_1282, B739_1283, B739_1284, B739_1285, B739_1286, B739_1287, B739_1288, B739_1289, B739_1290, B739_1291, B739_1292, B739_1293, B739_1294, B739_1295
1378278	1379182	+	3	B739_1296, B739_1297, B739_1298
1379338	1385150	+	9	B739_1299, B739_1300, B739_1301, B739_1302, B739_1303, B739_1304, B739_1305, B739_1306, B739_1307
1385239	1386525	-	4	B739_1308, B739_1309, B739_1310, B739_1311
1386917	1395409	+	3	B739_1312, B739_1313, B739_1314
1396921	1400003	-	2	B739_1316, B739_1317
1400194	1408340	+	8	B739_1318, B739_1319, B739_1320, B739_1321, B739_1322, B739_1323, B739_1324, B739_1325
1408443	1410082	+	2	B739_1326, B739_1327
1419830	1421033	-	2	B739_1335, B739_1336
1421221	1422975	+	2	B739_1337, B739_1338
1425956	1428911	-	2	B739_1342, B739_1343
1437924	1442270	-	2	B739_1351, B739_1352
1443632	1446624	+	4	B739_1354, B739_1355, B739_1356, B739_1357
1446648	1446982	-	2	B739_1358, B739_1359

1447268	1447778	-	2	B739_1360, B739_1361
1447911	1452801	+	10	B739_1362, B739_1363, B739_1364, B739_1365, B739_1366, B739_1367, B739_1368, B739_1369, B739_1370, B739_1371
1459328	1459980	-	2	B739_1375, B739_1376
1460010	1467226	+	8	B739_1377, B739_1378, B739_1379, B739_1380, B739_1381, B739_1382, B739_1383, B739_1384
1469685	1473242	-	3	B739_1386, B739_1387, B739_1388
1474538	1475410	-	2	B739_1390, B739_1391
1475602	1476419	+	2	B739_1392, B739_1393
1478280	1483780	+	4	B739_1395, B739_1396, B739_1397, B739_1398
1484381	1488787	+	5	B739_1400, B739_1401, B739_1402, B739_1403, B739_1404
1491477	1492808	+	2	B739_1407, B739_1408
1492985	1494689	-	2	B739_1409, B739_1410
1495208	1495838	+	2	B739_1411, B739_1412
1495941	1498901	+	2	B739_1413, B739_1414
1500046	1502773	+	2	B739_1416, B739_1417
1503263	1509043	+	3	B739_1418, B739_1419, B739_1420
1509208	1510045	+	2	B739_1421, B739_1422
1510850	1512975	-	3	B739_1425, B739_1426, B739_1427
1518519	1521145	-	3	B739_1431, B739_1432, B739_1433
1522908	1524539	-	2	B739_1435, B739_1436
1524791	1526861	+	2	B739_1437, B739_1438
1526894	1534886	-	2	B739_1439, B739_1440
1536512	1537972	+	3	B739_1443, B739_1444, B739_1445
1538198	1539000	+	2	B739_1446, B739_1447
1539149	1539829	+	2	B739_1448, B739_1449
1540094	1542429	+	4	B739_1450, B739_1451, B739_1452, B739_1453
1542892	1545101	-	3	B739_1455, B739_1456, B739_1457
1548595	1550809	+	2	B739_1462, B739_1463
1553573	1554039	-	2	B739_1469, B739_1470
1554239	1555712	-	2	B739_1471, B739_1472
1567169	1570195	+	4	B739_1484, B739_1485, B739_1486, B739_1487
1570225	1572103	-	3	B739_1488, B739_1489, B739_1490
1573299	1577503	+	6	B739_1492, B739_1493, B739_1494, B739_1495, B739_1496, B739_1497
1578855	1580410	+	2	B739_1499, B739_1500
1587806	1590250	+	3	B739_1506, B739_1507, B739_1508
1590223	1593372	-	3	B739_1509, B739_1510, B739_1511
1606143	1608282	+	2	B739_1525, B739_1526
1609434	1612106	+	4	B739_1528, B739_1529, B739_1530, B739_1531
1612243	1616658	+	6	B739_1532, B739_1533, B739_1534, B739_1535, B739_1536, B739_1537
1617125	1618804	+	2	B739_1538, B739_1539

1620480	1622375	+	2	B739_1541, B739_1542
1626097	1630086	-	2	B739_1547, B739_1548
1630313	1631988	-	2	B739_1549, B739_1550
1635815	1636484	+	2	B739_1553, B739_1554
1637307	1640589	-	2	B739_1556, B739_1557
1640877	1642577	-	2	B739_1558, B739_1559
1645332	1647125	+	3	B739_1562, B739_1563, B739_1564
1649674	1655373	+	4	B739_1568, B739_1569, B739_1570, B739_1571
1655806	1658233	-	3	B739_1573, B739_1574, B739_1575
1658261	1661944	+	4	B739_1576, B739_1577, B739_1578, B739_1579
1664920	1668775	-	5	B739_1582, B739_1583, B739_1584, B739_1585, B739_1586
1668926	1671554	-	3	B739_1587, B739_1588, B739_1589
1672079	1676495	+	3	B739_1590, B739_1591, B739_1592
1677342	1682046	+	4	B739_1594, B739_1595, B739_1596, B739_1597
1682318	1683115	-	2	B739_1598, B739_1599
1685099	1690047	+	6	B739_1602, B739_1603, B739_1604, B739_1605, B739_1606, B739_1607
1690210	1692916	+	3	B739_1608, B739_1609, B739_1610
1697123	1699146	+	2	B739_1613, B739_1614
1702529	1704327	+	2	B739_1616, B739_1617
1704429	1707414	+	2	B739_1618, B739_1619
1707683	1711651	-	3	B739_1620, B739_1621, B739_1622
1711719	1714219	+	2	B739_1623, B739_1624
1716209	1718581	+	2	B739_1626, B739_1627
1725245	1727802	-	2	B739_1633, B739_1634
1729400	1738749	-	8	B739_1636, B739_1637, B739_1638, B739_1639, B739_1640, B739_1641, B739_1642, B739_1643
1738978	1742162	-	6	B739_1644, B739_1645, B739_1646, B739_1647, B739_1648, B739_1649
1743807	1745046	-	2	B739_1652, B739_1653
1745510	1751103	-	7	B739_1655, B739_1656, B739_1657, B739_1658, B739_1659, B739_1660, B739_1661
1751375	1753424	-	2	B739_1662, B739_1663
1753587	1756397	-	4	B739_1664, B739_1665, B739_1666, B739_1667
1756508	1757886	-	2	B739_1668, B739_1669
1758041	1760272	-	4	B739_1670, B739_1671, B739_1672, B739_1673
1761110	1761965	-	3	B739_1675, B739_1676, B739_1677
1762193	1766415	-	3	B739_1678, B739_1679, B739_1680
1773818	1775227	+	2	B739_1686, B739_1687
1778178	1785384	+	7	B739_1689, B739_1690, B739_1691, B739_1692, B739_1693, B739_1694, B739_1695
1786766	1789594	+	2	B739_1697, B739_1698
1800030	1801478	-	3	B739_1706, B739_1707, B739_1705
1801584	1802755	-	2	B739_1709, B739_1710

1802874	1803702	-	2	B739_1718, B739_1719
1804051	1806646	-	3	B739_1720, B739_1721, B739_1722
1808291	1812084	+	2	B739_1724, B739_1725
1812148	1813412	-	2	B739_1726, B739_1727
1815152	1816867	+	2	B739_1730, B739_1731
1817558	1820820	+	4	B739_1733, B739_1734, B739_1735, B739_1736
1822344	1824498	+	4	B739_1738, B739_1739, B739_1740, B739_1741
1827686	1830895	+	3	B739_1745, B739_1746, B739_1747
1830990	1833869	-	4	B739_1748, B739_1749, B739_1750, B739_1751
1833945	1834569	+	2	B739_1752, B739_1753
1839977	1845237	+	5	B739_1760, B739_1761, B739_1762, B739_1763, B739_1764
1847768	1848716	-	2	B739_1766, B739_1767
1851012	1853186	+	2	B739_1770, B739_1771
1858601	1861108	-	4	B739_1777, B739_1778, B739_1779, B739_1780
1861237	1868573	-	5	B739_1781, B739_1782, B739_1783, B739_1784, B739_1785
1869179	1872705	+	5	B739_1787, B739_1788, B739_1789, B739_1790, B739_1791
1875138	1878367	-	3	B739_1795, B739_1796, B739_1797
1878593	1881458	-	3	B739_1798, B739_1799, B739_1800
1885069	1889446	+	2	B739_1803, B739_1804
1890407	1893358	-	3	B739_1805, B739_1806, B739_1807
1893638	1895933	+	3	B739_1808, B739_1809, B739_1810
1897440	1901008	-	8	B739_1812, B739_1813, B739_1814, B739_1815, B739_1816, B739_1817, B739_1818, B739_1819
1901677	1904450	+	4	B739_1820, B739_1821, B739_1822, B739_1823
1906165	1908113	-	3	B739_1826, B739_1827, B739_1828
1918047	1924012	-	3	B739_1839, B739_1840, B739_1841
1924306	1925413	-	2	B739_1842, B739_1843
1926002	1926733	-	2	B739_1845, B739_1846
1929114	1937727	+	12	B739_1848, B739_1849, B739_1850, B739_1851, B739_1852, B739_1853, B739_1854, B739_1855, B739_1856, B739_1857, B739_1858, B739_1859
1939688	1948308	-	5	B739_1861, B739_1862, B739_1863, B739_1864, B739_1865
1948520	1949100	+	2	B739_1866, B739_1867
1951067	1952269	+	2	B739_1869, B739_1870
1954500	1956989	-	3	B739_1872, B739_1873, B739_1874
1958482	1963127	-	3	B739_1877, B739_1878, B739_1879
1966130	1969497	+	2	B739_1881, B739_1882
1970040	1971512	-	2	B739_1884, B739_1885
1971615	1976057	-	2	B739_1886, B739_1887
1979864	1984400	-	2	B739_1890, B739_1891
1990398	1991999	-	2	B739_1896, B739_1897
2003936	2007359	+	2	B739_1904, B739_1905

2007410	2008590	-	2	B739_1906, B739_1907
2008808	2013566	-	2	B739_1908, B739_1909
2015361	2016983	-	2	B739_1911, B739_1912
2025859	2029560	+	4	B739_1923, B739_1924, B739_1925, B739_1926
2029600	2032036	-	2	B739_1927, B739_1928
2033461	2038744	-	6	B739_1930, B739_1931, B739_1932, B739_1933, B739_1934, B739_1935
2038961	2039715	-	2	B739_1936, B739_1937
2039982	2043878	-	5	B739_1938, B739_1939, B739_1940, B739_1941, B739_1942
2044390	2048297	+	3	B739_1944, B739_1945, B739_1946
2051023	2058025	+	7	B739_1949, B739_1950, B739_1951, B739_1952, B739_1953, B739_1954, B739_1955
2058155	2059350	+	2	B739_1956, B739_1957
2060707	2070030	-	7	B739_1959, B739_1960, B739_1961, B739_1962, B739_1963, B739_1964, B739_1965
2070135	2075115	-	8	B739_1966, B739_1967, B739_1968, B739_1969, B739_1970, B739_1971, B739_1972, B739_1973
2075105	2078993	+	5	B739_1974, B739_1975, B739_1976, B739_1977, B739_1978
2080497	2081444	+	2	B739_1980, B739_1981
2082328	2084036	-	5	B739_1983, B739_1984, B739_1985, B739_1986, B739_1987
2084463	2086269	-	5	B739_1989, B739_1990, B739_1991, B739_1992, B739_1993
2086473	2089978	-	3	B739_1994, B739_1995, B739_1996
2090472	2091769	-	4	B739_1997, B739_1998, B739_1999, B739_2000
2094882	2103813	-	4	B739_2005, B739_2006, B739_2007, B739_2008
2103980	2106584	-	4	B739_2009, B739_2010, B739_2011, B739_2012
2107518	2108286	-	2	B739_2015, B739_2016
2110440	2111000	+	2	B739_2019, B739_2020
2114928	2117296	-	3	B739_2022, B739_2023, B739_2024
2117437	2120722	+	2	B739_2025, B739_2026
2120734	2124535	-	4	B739_2027, B739_2028, B739_2029, B739_2030
2124667	2129698	-	2	B739_2031, B739_2032
2129930	2132879	+	2	B739_2033, B739_2034
2133945	2135828	-	2	B739_2037, B739_2038
2137107	2139686	-	2	B739_2040, B739_2041
2139826	2145049	+	6	B739_2042, B739_2043, B739_2044, B739_2045, B739_2046, B739_2047
2145073	2148430	-	4	B739_2048, B739_2049, B739_2050, B739_2051
2148593	2151699	+	4	B739_2052, B739_2053, B739_2054, B739_2055
2151718	2153937	-	3	B739_2056, B739_2057, B739_2058
2156813	2161344	-	4	B739_2062, B739_2063, B739_2064, B739_2065
2164404	2165644	-	2	B739_2069, B739_2070
2166913	2168337	-	2	B739_2072, B739_2073
2168482	2174635	+	6	B739_2074, B739_2075, B739_2076, B739_2077, B739_2078, B739_2079
2174632	2175829	-	2	B739_2080, B739_2081

2176473	2178772	-	2	B739_2082, B739_2083
2179036	2179357	+	2	B739_2084, B739_2085
2180240	2181881	-	3	B739_2087, B739_2088, B739_2089
2185491	2192068	-	3	B739_2091, B739_2092, B739_2093
2194901	2196820	-	3	B739_2097, B739_2098, B739_2099
2196976	2199709	-	3	B739_2100, B739_2101, B739_2102
2199810	2201866	+	3	B739_2103, B739_2104, B739_2105
2201904	2203636	-	2	B739_2106, B739_2107
2209021	2209844	+	2	B739_2110, B739_2111
2209859	2211009	-	2	B739_2112, B739_2113
2213140	2217241	-	2	B739_2116, B739_2117
2219223	2220113	+	2	B739_2119, B739_2120
2220110	2222098	-	3	B739_2121, B739_2122, B739_2123
2222252	2223605	+	2	B739_2124, B739_2125
2224017	2224650	+	2	B739_2127, B739_2128
2224833	2226164	+	2	B739_2129, B739_2130
2226322	2228273	-	3	B739_2131, B739_2132, B739_2133
2228421	2228909	-	2	B739_2134, B739_2135
2232541	2235298	+	2	B739_2139, B739_2140
2235680	2238616	+	3	B739_2141, B739_2142, B739_2143
2240677	2243517	-	2	B739_2147, B739_2148
2243833	2246219	-	2	B739_2149, B739_2150
2246314	2247460	-	2	B739_2151, B739_2152
2247580	2249446	-	2	B739_2153, B739_2154
2249520	2252072	-	3	B739_2155, B739_2156, B739_2157
2252207	2253509	+	2	B739_2158, B739_2159
2253658	2256671	+	2	B739_2160, B739_2161
2256875	2265044	+	6	B739_2162, B739_2163, B739_2164, B739_2165, B739_2166, B739_2167
2268097	2270440	-	2	B739_2169, B739_2170
2272190	2279106	+	8	B739_2174, B739_2175, B739_2176, B739_2177, B739_2178, B739_2179, B739_2180, B739_2181
2280583	2281573	+	2	B739_2183, B739_2184
2281714	2290046	+	8	B739_2185, B739_2186, B739_2187, B739_2188, B739_2189, B739_2190, B739_2191, B739_2192
2290118	2291490	-	2	B739_2193, B739_2194
2291602	2293397	-	3	B739_2195, B739_2196, B739_2197
2294198	2296956	-	3	B739_2199, B739_2200, B739_2201
	2299057	-	2	B739_2202, B739_2203
2301832	2304793	-	3	B739_2207, B739_2208, B739_2209
2306361	2306988	-	2	B739_2211, B739_2212

Table S3 Genes down-regulated in *Riemerella anatipestifer* CH-1 in iron-depleted conditions.

Gene ID	Genename	log2.Fold_change.	p-value	Description
13715110	B739_0004	-3.5536	8.39E-07	hypothetical protein
13715111	B739_0005	-1.1676	3.80E-05	phospholipid-binding protein
13715134	B739_0029	-1.2168	3.45E-13	Co/Zn/Cd efflux system protein
13715136	B739_0031	-2.1584	3.03E-07	Beta-lactamase class
13715137	B739_0032	-1.8951	7.80E-11	hypothetical protein
13715147	B739_0042	-1.0642	2.12E-12	hypothetical protein
13715148	B739_0043	-2.4394	6.03E-15	hypothetical protein
13715149	B739_0044	-2.6016	1.67E-13	hypothetical protein
13715150	B739_0045	-2.8968	1.17E-23	hypothetical protein
13715151	B739_0046	-2.9077	5.03E-20	hypothetical protein
13715152	B739_0047	-2.5115	4.98E-15	hypothetical protein
13715153	B739_0048	-2.1246	1.08E-32	hypothetical protein
13715154	B739_0049	-2.7847	8.28E-15	Beta-glucanase/Beta-glucan synthetase
13715155	B739_0050	-2.8906	2.36E-21	hypothetical protein
13715156	B739_0051	-2.0365	5.33E-14	hypothetical protein
13715157	B739_0052	-1.6093	1.26E-13	hypothetical protein
13715158	B739_0053	-2.0708	0.0028284	hypothetical protein
13715161	B739_0056	-1.9079	2.69E-19	Adenosylmethionine-8-amino-7-oxononanoate aminotransferase
13715162	B739_0057	-1.2533	5.18E-05	dethiobiotin synthetase
13715165	B739_0060	-1.4615	1.85E-09	hypothetical protein
13715166	B739_0061	-1.055	3.52E-10	Methionine synthase I, cobalamin-binding domain-containing protein
13715171	B739_0067	-4.2429	0.0045972	hypothetical protein
13715175	B739_0071	-2.6004	1.89E-22	hypothetical protein
13715180	B739_0076	-1.2584	7.39E-23	hypothetical protein
13715181	B739_0077	-1.1447	6.19E-31	hypothetical protein
13715182	B739_0078	-1.6657	1.08E-15	hypothetical protein
13715183	B739_0079	-1.3042	2.98E-16	hypothetical protein
13715184	B739_0080	-1.1427	8.20E-14	hypothetical protein
13715185	B739_0081	-1.26	2.78E-17	hypothetical protein
13715186	B739_0082	-1.3805	1.34E-39	MoxR-like ATPase
13715194	B739_0090	-2.0234	2.67E-12	hypothetical protein
13715195	B739_0091	-2.1064	1.61E-05	hypothetical protein
13715210	B739_0106	-1.2278	1.10E-13	amino acid transporter
13715219	B739_0115	-2.4021	4.38E-24	hypothetical protein
13715220	B739_0116	-2.6776	6.38E-15	hypothetical protein
13715221	B739_0117	-3.3195	5.54E-23	hypothetical protein

13715222	B739_0118	-3.0343	2.79E-10	hypothetical protein
13715228	B739_0124	-3.6112	4.42E-07	hypothetical protein
13715230	B739_0126	-1.3626	0.011918	hypothetical protein
13715234	B739_0130	-3.9687	0.01111	hypothetical protein
13715239	-//-	-1.2832	0.010073	
13715241	B739_0137	-2.372	0.0008521	hypothetical protein
13715242	B739_0138	-2.9944	8.25E-16	hypothetical protein
13715243	B739_0139	-3.0942	0.00089462	hypothetical protein
13715244	B739_0140	-2.6299	0.0057319	hypothetical protein
13715246	B739_0142	-2.5197	0.0090963	hypothetical protein
13715262	B739_0158	-1.1043	0.0010055	hypothetical protein
13715263	B739_0159	-1.1274	9.66E-05	hypothetical protein
13715273	B739_0169	-1.0421	1.90E-49	stress-responsive transcriptional regulator
13715287	B739_0183	-1.3845	4.24E-05	hypothetical protein
13715288	B739_0184	-1.7237	0.0022932	hypothetical protein
13715290	B739_0186	-3.1274	9.26E-06	hypothetical protein
13715291	B739_0187	-2.8206	1.19E-27	hypothetical protein
13715292	B739_0188	-2.7019	7.24E-05	hypothetical protein
13715293	B739_0189	-2.3067	0.0056787	hypothetical protein
13715294	B739_0190	-2.0762	5.10E-08	hypothetical protein
13715295	B739_0191	-2.4565	4.13E-06	hypothetical protein
13715296	B739_0192	-2.8836	6.67E-09	hypothetical protein
13715300	B739_2167	-1.2016	2.48E-12	hypothetical protein
13715305	B739_2172	-2.8326	2.27E-05	hypothetical protein
13715326	B739_2193	-1.8957	5.22E-07	hypothetical protein
13715327	B739_2194	-2.4037	1.91E-11	hypothetical protein
13715328	B739_2195	-1.2439	0.00063856	hypothetical protein
13715329	B739_2196	-1.2511	0.0065652	hypothetical protein
13715332	B739_2199	-3.5295	7.53E-20	hypothetical protein
13715333	B739_2200	-2.3811	0.011733	GTPase subunit of restriction endonuclease
13715335	B739_2202	-2.1686	2.58E-07	hypothetical protein
13715336	B739_2203	-1.4753	9.34E-07	hypothetical protein
13715337	B739_2204	-2.2529	0.0001849	hypothetical protein
13715338	B739_2205	-2.9347	2.16E-11	hypothetical protein
13715339	B739_2206	-1.813	5.47E-06	hypothetical protein
13715340	B739_2207	-2.7648	0.010402	hypothetical protein
13715371	B739_1538	-1.3763	1.59E-64	hypothetical protein
13715396	B739_0855	-1.1762	2.05E-12	NAD-dependent aldehyde dehydrogenase
13715415	B739_0874	-2.4597	3.04E-11	hypothetical protein
13715416	B739_0875	-2.7261	5.59E-15	hypothetical protein
13715417	B739_0876	-2.7217	1.24E-40	hypothetical protein

13715419	B739_0878	-2.5032	3.13E-09	hypothetical protein
13715423	B739_0882	-1.5611	1.12E-06	hypothetical protein
13715435	B739_0894	-1.1395	3.15E-12	hypothetical protein
13715442	B739_0901	-1.1851	3.92E-15	Fe-S oxidoreductase
13715446	B739_0906	-3.4672	0.00079524	hypothetical protein
13715447	B739_0907	-1.8975	1.13E-19	hypothetical protein
13715448	B739_0908	-1.633	1.80E-08	hypothetical protein
13715449	B739_0909	-1.7075	4.63E-11	hypothetical protein
13715456	B739_0916	-1.3801	1.52E-10	hydrolase
13715467	B739_0927	-1.9127	9.71E-160	Trypsin-like serine protease
13715478	-//-	-2.1179	0.00020597	
13715483	B739_0943	-3.066	7.51E-12	hypothetical protein
13715486	B739_0946	-1.881	4.26E-38	Formate-dependent nitrite reductase, periplasmic cytochrome c552 subunit
13715487	B739_0947	-2.0873	1.04E-17	hypothetical protein
13715488	B739_0948	-2.0582	7.91E-18	ABC transporter involved in cytochrome c biogenesis permease
13715489	B739_0949	-1.1374	5.15E-13	flavoprotein
13715504	B739_0964	-2.7053	2.25E-27	hypothetical protein
13715550	B739_1011	-1.6084	4.09E-17	hypothetical protein
13715551	B739_1012	-1.1952	2.59E-09	hypothetical protein
13715552	B739_1013	-1.018	7.33E-06	hypothetical protein
13715554	B739_1015	-1.3212	3.27E-12	D-alanine export protein
13715555	B739_1016	-1.6913	5.17E-09	hypothetical protein
13715556	B739_1017	-1.7258	5.48E-12	hypothetical protein
13715560	B739_1021	-1.2135	3.35E-28	sulfate permease
13715576	B739_1037	-1.669	1.38E-07	hypothetical protein
13715580	B739_1041	-4.7584	2.27E-09	hypothetical protein
13715583	B739_1044	-2.1228	3.84E-14	hypothetical protein
13715584	B739_1045	-2.0307	1.46E-32	hypothetical protein
13715588	B739_1049	-1.3234	1.20E-11	hypothetical protein
13715596	B739_1057	-1.3895	9.44E-14	hypothetical protein
13715624	B739_1086	-1.0298	6.17E-05	hypothetical protein
13715625	B739_1087	-1.6038	0.0032817	hypothetical protein
13715632	B739_1095	-1.6967	1.75E-20	hypothetical protein
13715636	B739_1099	-1.2696	3.50E-11	nucleotidyltransferase/DNA polymerase
13715646	B739_1109	-2.3241	2.79E-09	hypothetical protein
13715647	B739_1110	-2.7241	2.40E-20	hypothetical protein
13715654	B739_1117	-1.1095	2.74E-05	intracellular protease/amidase
13715672	B739_1135	-1.0991	8.89E-06	Acetyltransferase (isoleucine patch superfamily)
13715674	B739_1137	-1.3933	1.03E-07	hypothetical protein

13715684	B739_1147	-1.787	8.23E-09	hypothetical protein
13715705	B739_1168	-1.2944	8.80E-06	DnaJ-class molecular chaperone with C-terminal Zn finger domain
13715714	B739_1177	-1.6766	9.81E-18	hypothetical protein
13715729	B739_1192	-1.7563	2.13E-11	hypothetical protein
13715761	B739_1224	-2.2439	1.11E-11	hypothetical protein
13715762	B739_1225	-1.5609	5.62E-22	hypothetical protein
13715763	B739_1226	-2.01	0.0014052	hypothetical protein
13715764	B739_1227	-1.5237	0.00073354	hypothetical protein
13715766	B739_1229	-2.3307	2.78E-05	hypothetical protein
13715799	B739_1262	-1.4493	0.010119	hypothetical protein
13715803	B739_1266	-1.3794	8.30E-22	hypothetical protein
13715804	B739_1267	-2.6128	0.00040716	hypothetical protein
13715807	B739_1270	-3.9347	0.0003978	hypothetical protein
13715808	B739_1271	-2.6256	4.83E-07	hypothetical protein
13715814	B739_1277	-2.6192	0.0015104	hypothetical protein
13715815	B739_1278	-3.351	1.97E-24	hypothetical protein
13715819	B739_1282	-3.7597	0.0026423	hypothetical protein
13715821	B739_1284	-2.1335	3.05E-06	hypothetical protein
13715822	B739_1285	-2.9313	1.09E-35	hypothetical protein
13715823	B739_1286	-1.894	1.95E-06	hypothetical protein
13715824	B739_1287	-1.8411	0.011071	hypothetical protein
13715825	B739_1288	-2.0381	0.0040908	hypothetical protein
13715827	B739_1290	-1.9899	0.0089049	hypothetical protein
13715828	B739_1291	-2.1669	0.0012845	hypothetical protein
13715829	B739_1292	-2.8506	1.10E-22	hypothetical protein
13715835	B739_1298	-1.0132	0.0096976	hypothetical protein
13715839	B739_1302	-2.668	9.29E-21	hypothetical protein
13715841	B739_1304	-1.9679	0.00030863	hypothetical protein
13715842	B739_1305	-2.4695	1.38E-11	hypothetical protein
13715843	B739_1306	-1.8823	0.01061	hypothetical protein
13715844	B739_1307	-2.9016	1.36E-09	hypothetical protein
13715845	B739_1308	-2.6788	1.06E-05	hypothetical protein
13715847	B739_1310	-3.1274	0.002277	hypothetical protein
13715848	B739_1311	-2.4733	3.15E-45	hypothetical protein
13715849	B739_1312	-3.4338	8.63E-94	hypothetical protein
13715850	B739_1313	-3.1626	6.01E-23	hypothetical protein
13715851	B739_1314	-2.3107	9.82E-22	hypothetical protein
13715869	B739_1332	-1.6531	3.25E-35	hypothetical protein
13715870	B739_1333	-1.2401	2.58E-10	hypothetical protein
13715872	B739_1335	-2.2125	0.00010926	hypothetical protein

13715873	B739_1336	-1.0387	4.57E-05	hypothetical protein
13715880	B739_1343	-1.1098	1.45E-12	hypothetical protein
13715883	B739_1346	-3.562	7.66E-07	recombinase A
13715885	B739_1348	-2.5948	2.94E-30	hypothetical protein
13715890	B739_1353	-3.3628	1.88E-20	hypothetical protein
13715891	B739_1354	-3.2996	6.06E-20	hypothetical protein
13715892	B739_1355	-3.7338	9.90E-17	hypothetical protein
13715894	B739_1357	-1.329	5.56E-05	hypothetical protein
13715899	B739_1362	-2.7053	1.35E-06	hypothetical protein
13715900	B739_1363	-3.1678	1.17E-06	hypothetical protein
13715901	B739_1364	-2.7344	1.19E-11	hypothetical protein
13715903	B739_1366	-3.5722	0.0001959	hypothetical protein
13715904	B739_1367	-3.6922	5.98E-06	hypothetical protein
13715905	B739_1368	-3.2567	0.0022636	hypothetical protein
13715906	B739_1369	-2.1274	1.26E-05	hypothetical protein
13715907	B739_1370	-2.6364	0.0039116	hypothetical protein
13715908	B739_1371	-3.0816	0.0048619	hypothetical protein
13715911	B739_1374	-3.0951	1.95E-83	hypothetical protein
13715914	B739_1377	-2.6405	8.55E-05	hypothetical protein
13715915	B739_1378	-3.4779	6.74E-11	hypothetical protein
13715916	B739_1379	-3.1461	2.63E-52	hypothetical protein
13715919	B739_1382	-2.802	9.16E-06	hypothetical protein
13715920	B739_1383	-3.2914	1.21E-08	hypothetical protein
13715930	B739_1393	-1.0897	0.00031744	D-Tyr-tRNAtyr deacylase
13715931	B739_1394	-1.137	4.53E-24	Periplasmic serine proteases (ClpP class)
13715958	B739_1421	-1.6644	0.0012459	hypothetical protein
13715960	B739_1423	-3.1497	4.78E-07	hypothetical protein
13715974	B739_1437	-1.51	2.74E-05	hypothetical protein
13715975	B739_1438	-2.0672	2.54E-09	hypothetical protein
13715978	B739_1441	-1.793	0.00017615	hypothetical protein
13715980	B739_1443	-2.5703	1.22E-05	hypothetical protein
13716001	B739_1468	-2.7554	0.0048132	hypothetical protein
13716002	B739_1469	-3.8532	4.64E-07	hypothetical protein
13716020	B739_1488	-1.0669	7.60E-05	Dihydropteroate synthase-related enzyme
13716037	B739_1505	-1.8344	4.44E-08	hypothetical protein
13716048	B739_1010	-2.1274	0.0055268	hypothetical protein
13716060	B739_1529	-2.2598	1.99E-17	hypothetical protein
13716062	B739_1531	-1.8119	0.0083288	hypothetical protein
13716093	B739_1562	-1.3651	6.55E-68	Superfamily II DNA and RNA helicase
13716094	B739_1563	-1.5602	7.86E-17	Cold shock protein
13716095	B739_1564	-4.2436	0	Cold shock protein

13716101	B739_1570	-1.0399	0.00062784	hypothetical protein
13716104	B739_1573	-1.8452	1.44E-14	hypothetical protein
13716113	B739_1582	-2.5246	1.04E-13	molybdopterin and thiamine biosynthesis family protein
13716114	B739_1583	-2.7305	8.56E-14	thiamine biosynthesis enzyme ThiH-related protein
13716115	B739_1584	-2.4116	2.20E-08	enzyme of thiazole biosynthesis
13716116	B739_1585	-2.5263	2.68E-09	hypothetical protein
13716118	B739_1587	-1.5775	0.00033613	hypothetical protein
13716119	B739_1588	-1.7398	2.99E-22	Thiamine biosynthesis protein ThiC
13716129	-//-	-1.4789	0.0025438	
13716147	B739_1616	-1.3843	6.09E-05	hypothetical protein
13716163	B739_1632	-2.8337	0.00011709	hypothetical protein
13716167	B739_1636	-2.8416	1.04E-23	hypothetical protein
13716168	B739_1637	-2.663	2.47E-17	hypothetical protein
13716170	B739_1639	-3.0479	4.34E-31	hypothetical protein
13716171	B739_1640	-2.0194	4.24E-05	hypothetical protein
13716181	B739_1650	-2.5628	0.00030303	hypothetical protein
13716182	B739_1651	-2.036	1.49E-05	hypothetical protein
13716184	B739_1653	-2.9953	0.0025074	hypothetical protein
13716187	B739_1657	-2.4452	1.41E-09	hypothetical protein
13716188	B739_1658	-2.3057	0.0034741	hypothetical protein
13716189	B739_1659	-2.578	2.11E-05	hypothetical protein
13716190	B739_1660	-2.9854	4.35E-15	hypothetical protein
13716191	B739_1661	-2.4493	0.00038082	hypothetical protein
13716192	B739_1662	-3.5093	9.82E-23	hypothetical protein
13716193	B739_1663	-2.8676	2.50E-05	hypothetical protein
13716194	B739_1664	-2.7865	2.27E-11	DNA modification methylase
13716195	B739_1665	-2.9782	0.00038934	hypothetical protein
13716198	B739_1668	-2.2703	5.27E-06	hypothetical protein
13716199	B739_1669	-1.4856	0.0031414	hypothetical protein
13716200	B739_1670	-2.6093	2.14E-05	hypothetical protein
13716201	B739_1671	-3.36	5.84E-10	hypothetical protein
13716209	B739_1679	-2.1873	2.11E-20	hypothetical protein
13716225	B739_1695	-1.2877	4.67E-07	hypothetical protein
13716280	B739_1757	-2.6658	1.40E-07	ssDNA-binding protein
13716291	B739_1768	-2.5748	0.010149	hypothetical protein
13716305	B739_1782	-1.1631	3.12E-23	hypothetical protein
13716306	B739_1783	-1.1149	2.35E-19	hypothetical protein
13716307	B739_1784	-1.1053	7.82E-21	hypothetical protein
13716308	B739_1785	-1.6252	2.62E-123	silver efflux pump

13716316	B739_1793	-1.5134	3.02E-06	hypothetical protein
13716317	B739_1794	-2.5623	1.53E-08	hypothetical protein
13716318	B739_1795	-2.48	8.70E-24	membrane-bound metal-dependent hydrolase
13716319	B739_1796	-3.773	2.85E-27	hypothetical protein
13716320	B739_1797	-3.6579	0.00011165	hypothetical protein
13716321	B739_1798	-3.6509	4.89E-05	hypothetical protein
13716322	B739_1799	-3.0816	2.21E-16	hypothetical protein
13716336	B739_1813	-1.1215	0.001017	hypothetical protein
13716341	B739_1818	-1.2176	0.010983	hypothetical protein
13716352	B739_1829	-1.3284	4.31E-18	hypothetical protein
13716359	B739_1836	-3.6128	1.61E-08	protocatechuate 3,4-dioxygenase subunit beta
13716380	B739_1857	-1.1941	0.0064467	hypothetical protein
13716382	B739_1859	-1.2603	0.011539	hypothetical protein
13716394	B739_1871	-1.7265	3.96E-13	hypothetical protein
13716412	B739_1890	-1.2994	5.90E-39	hypothetical protein
13716413	B739_1891	-1.2575	7.93E-10	hypothetical protein
13716427	B739_1905	-1.341	0.00054007	hypothetical protein
13716479	B739_1958	-2.9575	3.04E-10	hypothetical protein
13716480	B739_1959	-3.2359	0.0024902	hypothetical protein
13716482	B739_1961	-3.0135	3.37E-07	hypothetical protein
13716483	B739_1962	-3.4304	3.23E-22	hypothetical protein
13716484	B739_1963	-3.3073	9.96E-06	hypothetical protein
13716485	B739_1964	-3.6645	1.53E-70	hypothetical protein
13716487	B739_1966	-2.905	0.00061683	hypothetical protein
13716488	B739_1967	-2.8823	1.67E-06	hypothetical protein
13716489	B739_1968	-3.982	1.14E-08	hypothetical protein
13716490	B739_1969	-2.6098	0.0011365	hypothetical protein
13716491	B739_1970	-3.1529	0.006464	hypothetical protein
13716492	B739_1971	-3.9347	1.44E-05	Negative regulator of beta-lactamase expression
13716493	B739_1972	-3.3753	8.35E-12	hypothetical protein
13716494	B739_1973	-3.73	5.20E-12	hypothetical protein
13716495	B739_1974	-3.1014	0.0078436	hypothetical protein
13716496	B739_1975	-2.7998	0.0005084	hypothetical protein
13716497	B739_1976	-3.756	1.28E-16	hypothetical protein
13716498	B739_1977	-3.3577	4.63E-05	hypothetical protein
13716499	B739_1978	-3.6312	2.27E-24	hypothetical protein
13716500	B739_1979	-2.9027	4.35E-11	hypothetical protein
13716501	B739_1980	-2.615	0.00014724	hypothetical protein
13716502	B739_1981	-2.5243	3.56E-05	hypothetical protein
13716505	B739_1984	-3.4128	3.63E-06	hypothetical protein
13716507	B739_1986	-2.5099	0.0050666	hypothetical protein

13716510	B739_1989	-3.1542	4.11E-06	hypothetical protein
13716511	B739_1990	-2.9129	6.22E-05	hypothetical protein
13716515	B739_1994	-3.7322	7.44E-11	hypothetical protein
13716516	B739_1995	-3.043	1.95E-08	hypothetical protein
13716517	B739_1996	-3.5295	5.75E-29	hypothetical protein
13716519	B739_1998	-3.8823	0.00052834	hypothetical protein
13716520	B739_1999	-3.6142	3.59E-08	prophage antirepressor
13716523	B739_2002	-2.7356	7.08E-08	hypothetical protein
13716525	B739_2004	-1.9288	0.0014865	hypothetical protein
13716576	B739_2055	-1.644	1.48E-12	hypothetical protein
13716580	B739_2059	-2.2006	0.0019689	hypothetical protein
13716664	B739_2143	-1.0556	0.0011685	hypothetical protein
13716688	B739_0196	-2.6705	1.45E-06	hypothetical protein
13716689	B739_0197	-1.0527	5.49E-05	hypothetical protein
13716692	B739_0200	-1.5252	1.81E-46	hypothetical protein
13716696	B739_0204	-1.1612	1.23E-07	hypothetical protein
13716698	B739_0207	-2.6881	0.00023828	hypothetical protein
13716702	B739_0211	-3.6192	1.47E-08	hypothetical protein
13716705	B739_0214	-1.5777	3.62E-10	Signal transduction histidine kinase
13716707	B739_0216	-2.7848	1.46E-19	hypothetical protein
13716708	B739_0217	-2.8564	7.40E-24	hypothetical protein
13716736	B739_0245	-1.1335	3.69E-06	hypothetical protein
13716737	B739_0246	-1.3404	0.0037566	hypothetical protein
13716761	B739_0270	-1.1904	1.62E-50	hypothetical protein
13716762	B739_0271	-3.4062	1.94E-06	hypothetical protein
13716764	B739_0274	-2.0411	1.67E-05	hypothetical protein
13716766	B739_0275	-2.8628	7.94E-10	hypothetical protein
13716767	B739_0276	-3.7299	1.33E-30	hypothetical protein
13716768	B739_0277	-2.1189	9.35E-12	Membrane protein related to metalloendopeptidase
13716769	B739_0278	-1.112	1.19E-18	hypothetical protein
13716770	B739_0279	-4.1004	9.41E-16	hypothetical protein
13716771	B739_0280	-1.4493	4.78E-05	hypothetical protein
13716774	B739_0283	-1.8959	1.07E-06	hypothetical protein
13716811	B739_0321	-2.5368	0.0023722	hypothetical protein
13716819	B739_0329	-1.4034	7.18E-21	hypothetical protein
13716843	B739_0355	-1.9249	3.81E-12	hypothetical protein
13716877	B739_0389	-2.298	2.32E-25	hypothetical protein
13716878	B739_0390	-1.5627	1.98E-09	hypothetical protein
13716888	B739_0400	-1.1837	2.37E-06	hypothetical protein
13716894	B739_0406	-1.6047	3.21E-32	hypothetical protein
13716942	B739_0454	-1.0246	0.00056591	site-specific recombinase XerD

13716943	B739_0455	-1.2138	0.0037585	hypothetical protein
13716948	B739_0460	-1.3951	9.00E-12	hypothetical protein
13716951	B739_0463	-1.0926	0.0029649	hypothetical protein
13716959	B739_0471	-1.1398	4.34E-06	hypothetical protein
13716972	B739_0484	-1.5077	5.54E-06	hypothetical protein
13716973	B739_0485	-1.6632	1.52E-09	hypothetical protein
13716975	B739_0487	-1.6252	1.24E-22	hypothetical protein
13716976	B739_0488	-1.4465	8.62E-05	hypothetical protein
13716977	B739_0489	-3.0534	0.00014169	hypothetical protein
13716982	B739_0494	-1.4308	1.58E-07	Histone acetyltransferase HPA2-related acetyltransferase
13716993	B739_0505	-1.5253	5.71E-18	hypothetical protein
13716994	B739_0506	-1.3384	1.05E-12	hypothetical protein
13717004	B739_0516	-1.5868	5.87E-07	hypothetical protein
13717009	B739_0521	-1.626	4.24E-07	hypothetical protein
13717026	B739_0538	-3.0181	5.28E-37	hypothetical protein
13717027	B739_0539	-3.4169	0.00050172	hypothetical protein
13717043	B739_0555	-1.2931	0.00022915	hypothetical protein
13717050	B739_0562	-1.0826	8.13E-16	hypothetical protein
13717079	-//-	-2.1274	0.010205	
13717084	B739_0596	-1.9018	1.72E-08	hypothetical protein
13717090	B739_0602	-1.5164	1.29E-07	Rhodanese-related sulfurtransferase
13717104	B739_0616	-1.1874	9.81E-05	response regulator containing CheY-like receiver and SARP domains
13717105	B739_0617	-1.3984	4.89E-06	hypothetical protein
13717106	B739_0618	-1.0044	0.00093961	hypothetical protein
13717121	B739_0633	-2.1753	0.0001524	hypothetical protein
13717122	B739_0634	-1.3709	0.00065609	hypothetical protein
13717124	B739_0636	-1.6516	8.19E-65	hypothetical protein
13717133	B739_0645	-1.1337	5.62E-06	hypothetical protein
13717141	B739_0653	-2.6141	1.26E-18	hypothetical protein
13717160	B739_0672	-1.6689	6.45E-100	hypothetical protein
13717179	B739_0691	-1.7423	2.94E-10	hypothetical protein
13717189	B739_0702	-1.656	1.52E-13	hypothetical protein
13717190	B739_0703	-1.3893	5.17E-08	hypothetical protein
13717196	B739_0710	-1.8055	4.09E-12	hypothetical protein
13717201	B739_0715	-1.7264	0.0059863	hypothetical protein
13717202	B739_0716	-1.9954	9.07E-13	hypothetical protein
13717212	B739_0726	-1.5349	5.60E-06	hypothetical protein
13717213	B739_0727	-2.3046	2.65E-12	Zn-dependent hydrolase, including glyoxylase
13717221	B739_0735	-2.9087	0.0095116	hypothetical protein

13717253	B739_0767	-2.0287	2.41E-21	hypothetical protein
13717254	B739_0768	-1.0074	3.83E-10	hypothetical protein
13717255	B739_0769	-2.7152	3.21E-14	hypothetical protein
13717263	B739_0777	-1.2289	0.0014001	hypothetical protein
13717266	B739_0780	-1.6812	1.81E-34	hypothetical protein
13717267	B739_0781	-1.7733	3.37E-264	Cation/multidrug efflux pump
13717268	B739_0782	-1.5725	3.33E-32	hypothetical protein
13717278	B739_0792	-1.1293	2.91E-23	aminopeptidase
13717288	B739_0802	-1.3634	1.32E-07	cytosine/adenosine deaminase
13717293	B739_0807	-1.1611	3.35E-19	hypothetical protein
13717302	B739_0817	-4.0581	0.0023634	hypothetical protein
13717303	B739_0818	-2.8375	3.26E-21	hypothetical protein
13717304	B739_0819	-2.6211	2.06E-20	hypothetical protein
13717305	B739_0820	-2.4038	7.07E-08	hypothetical protein
13717324	B739_0839	-1.0726	1.19E-09	acyltransferase
